# Peroxisomal integrity in demyelination-associated microglia enables cellular debris clearance and myelin renewal in mice

**DOI:** 10.1172/JCI179985

**Published:** 2025-11-06

**Authors:** Joseph A. Barnes-Vélez, Xiaohong Zhang, Yaren L. Peña Señeriz, Kiersten A. Scott, Yinglu Guan, Jian Hu

**Affiliations:** 1 Department of Cancer Biology, MD Anderson Cancer Center, Houston, Texas, USA.; 2 University of Texas MD Anderson Cancer Center UTHealth Graduate School of Biomedical Sciences, Houston, Texas, USA,; 3 University of Puerto Rico School of Medicine, San Juan, Puerto Rico.; 4 University of Puerto Rico at Cayey, Cayey, Puerto Rico.; 5 Cancer Neuroscience Program, MD Anderson Cancer Center, Houston, Texas, USA.

**Keywords:** Cell biology, Inflammation, Neuroscience, Demyelinating disorders, Macrophages

## Abstract

Demyelination associated microglia (DMAM) orchestrate the regenerative response to demyelination by clearing myelin debris and promoting oligodendrocyte maturation. Peroxisomal metabolism has emerged as a candidate regulator of DMAMs, though the cell-intrinsic contribution in microglia remains undefined. Here we elucidate the role of peroxisome integrity in DMAMs, using cuprizone-mediated demyelination coupled with conditional KO of peroxisome biogenesis factor 5 (PEX5) in microglia. Absent demyelination, PEX5 conditional KO (PEX5cKO) had minimal impact on homeostatic microglia. However, during cuprizone-induced demyelination, the emergence of DMAMs unmasked a critical requirement for peroxisome integrity. At peak demyelination, PEX5cKO DMAMs exhibited increased lipid droplet burden and reduced lipophagy suggestive of impaired lipid catabolism. Although lipid droplet burden declined during the remyelination phase, PEX5cKO DMAMs accumulated intralysosomal crystals and curvilinear profiles, features that were largely absent in controls. Aberrant lipid processing was accompanied by elevated numbers of lysosomal damage markers and downregulation of the lipid exporter gene *Apoe*, consistent with defective lipid clearance. Furthermore, the disruptions in PEX5cKO DMAMs were associated with defective myelin debris clearance and impaired remyelination. Together, these findings delineate a stage-specific role for peroxisomes in coordinating lipid processing pathways essential to DMAM function and for enabling a pro-remyelinating environment.

## Abstract 2

Loss of peroxisomal integrity disrupts myelin debris-derived lipid processing in demyelination associated microglia and undermines myelin debris clearance and remyelination.

## Graphical abstract

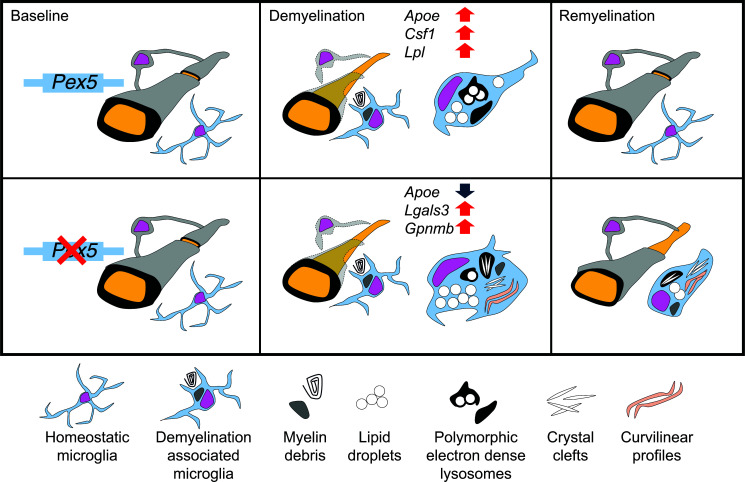

## Introduction

Peroxisomes are single-membrane-bound organelles required for a subset of metabolic pathways, including ether lipid synthesis and very-long-chain fatty acid oxidation (1). Recently, evidence linking peroxisomes to immunometabolism (2) has been described in macrophages, wherein through cell intrinsic mechanisms peroxisomes facilitate pathogen clearance (3), cytokine release (4), and bioactive lipid-precursor mobilization (5). Moreover, peroxisomal lipid oxidation regulates adaptations to age-related stress in microglia, the predominant subset of brain resident macrophages (6, 7). Microglia drive a critical response to demyelination by clearing myelin debris and enabling newly formed oligodendrocytes to remyelinate denuded axons (8). Studies of peroxisomal disease have suggested that peroxisomes also contribute to demyelination associated microglia (DMAMs) and the microglial response in demyelination. For instance, in adrenoleukodystrophy, germline mutations disrupting peroxisomal lipid oxidation can precipitate demyelination associated with perilesional microglial stress and loss (9, 10). However, whether peroxisomes facilitate DMAMs cell autonomously and to what extent remain unclear.

To ascertain the cell-intrinsic role of peroxisomes in DMAMs, we disrupted peroxisome biogenesis in microglia by conditional depletion of PEX5 (encoded by *Pex5*) within the context of demyelination induced by cuprizone (CPZ). PEX5 loss redirected microglial activation into DMAMs enriched for lysosome-damage-related genes *Lgals3* and *Gpnmb*. In contrast to peroxisome-intact DMAMs, lipid droplet clearance in PEX5cKO DMAMs correlated with notable crystal accumulation and loss of lipophagy-invoking structures. Moreover, PEX5 depletion in DMAMs reduced myelin debris clearance and pro-remyelination factor expression, which correlated with impaired remyelination. In summary, the microglial response during demyelination is intrinsically dependent on peroxisomal integrity for acquiring a pro-remyelinating phenotype.

## Results

### Validation of PEX5 depletion in myeloid cells.

We asked whether microglia intrinsically depend on peroxisomes to facilitate a response to demyelination. To address this question, we achieved PEX5cKO using *Cx3cr1*^CreERT2/wt^, *Pex5*^lox/lox^ mice (11, 12), in which tamoxifen-inducible Cre recombinase drives PEX5 depletion in myeloid cells, including microglia. *Cx3Cr1*^CreERT2/wt^, *Pex5*^lox/wt,wt/wt^ littermates served as controls. In vitro, 4-hydroxytamoxifen exposure downregulated both *Pex5* mRNA and PEX5 protein in PEX5cKO bone marrow–derived macrophages relative to controls (Supplemental Figure 1, A–C; supplemental material available online with this article; https://doi.org/10.1172/JCI179985DS1). Because PEX5 loss undermines peroxisome biogenesis (13), we examined peroxisome integrity. Immunolabeling for peroxisome membrane protein 70 (PMP70) revealed that PEX5cKO macrophages, relative to controls, had fewer and larger PMP70^+^ punctae, suggestive of enlarged ghost peroxisomes or peroxisomal clumping (Supplemental Figure 1, D–F) indicative of disrupted peroxisome homeostasis (13).

### Experimental design to assess the role of PEX5 in DMAMs.

To evaluate the effects of PEX5 loss in DMAMs, we fed adult PEX5cKO and control littermates a 0.2% CPZ diet for 6 weeks (Supplemental Figure 1G). Two weeks prior to starting CPZ, both PEX5cKO and control littermates received tamoxifen (2 mg daily, 5 consecutive days) to induce PEX5 KO and enable turnover of healthy peroxisomes to dysfunctional peroxisomes (14). After 6 weeks of CPZ, brain samples were collected to assess peak demyelination. Baseline condition consisted of PEX5cKO and control littermates fed standard chow throughout the study (Supplemental Figure 1G). Whole-brain samples were collected and submitted to single-nuclei RNA-Seq (snRNA-Seq) for transcriptional profiling of the cell types of interest.

### PEX5 loss diverges DMAM evolution after demyelination.

**Figure 1:**
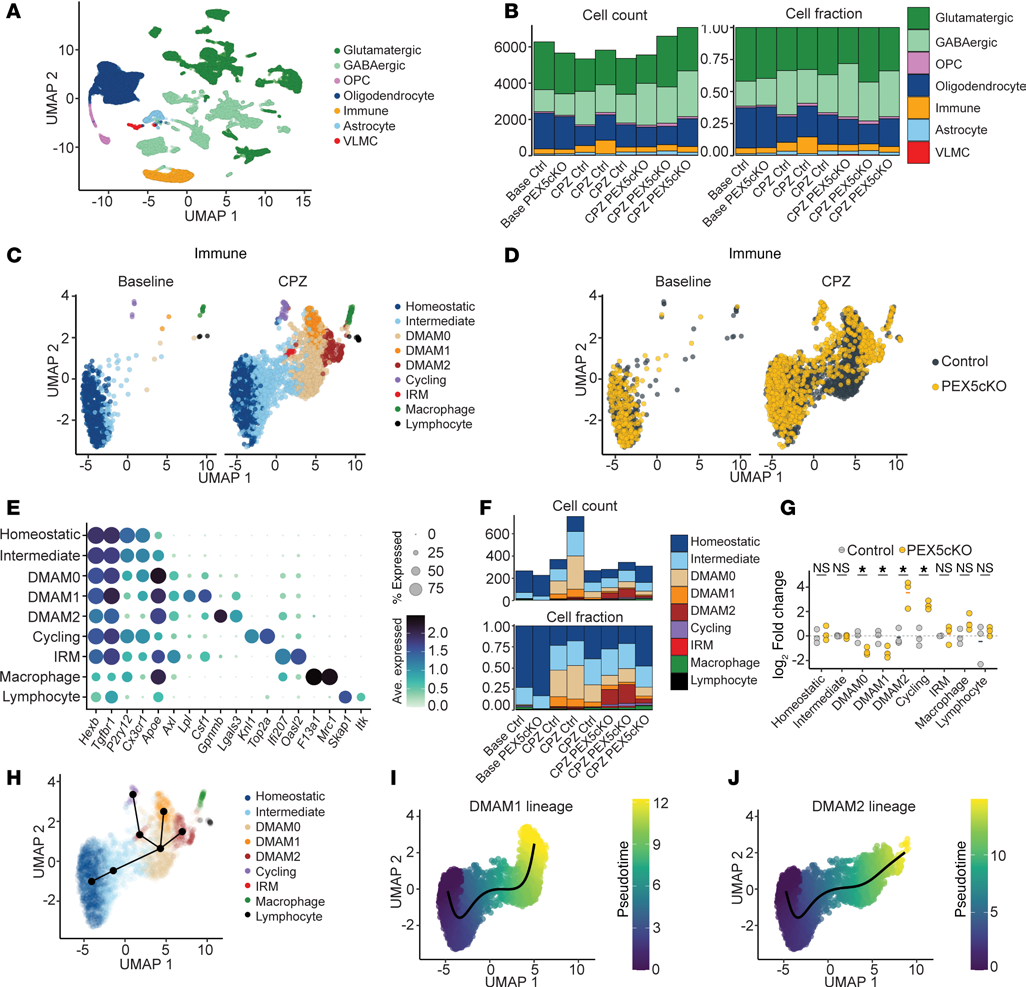
DMAM2 cluster emerges from PEX5cKO microglia after demyelination. (**A**) Uniform manifold approximation and projection (UMAP) of combined whole-brain nuclei detected from baseline and CPZ conditions colored according to major cell-type identity. (**B**) Cell count and cell fraction per major cell type identified in baseline and CPZ conditions across PEX5cKO and control (Ctrl) samples. (**C**) UMAP of immune subclusters colored according to subcluster identity and split by conditions. (**D**) UMAP of immune subclusters colored according to genotype and split by condition. (**E**) Average gene expression of marker genes per immune subcluster. Dot color and size correspond to average expression and percentage expressed, respectively. (**F**) Cell count and cell fraction per immune subcluster identified within each library generated from baseline (*n* = 1 per genotype) and CPZ (*n* = 3 per genotype) conditions. (**G**) Cell fraction log_2_ fold change relative to control per immune subcluster. Individual data points correspond to libraries generated from the CPZ condition. Bars correspond to the mean log_2_ fold change. *P < 0.05, unpaired, 2-tailed *t* test. (**H**) Inferred lineages for microglial subclusters derived from slingshot analysis superimposed onto immune cell UMAP, with the homeostatic subcluster designated as the origin cluster. (**I**) Pseudotime trajectory for DMAM1 lineage. (**J**) Pseudotime trajectory for DMAM2 lineage. Ave., average; IRM, interferon-responsive microglia; VLMC, vascular and leptomeningeal cell.

After quality control, dimensionality reduction, cell clustering, and cell marker–based annotation (Supplemental Figure 2, A–C, and Supplemental Table 1), we detected 29 cell clusters, 24 of which were neuronal (*Syt1* enriched) and further categorized into glutamatergic (expressing *Slc17a7* and *Slc17a6*) and GABAergic (expressing *Gad1* and *Gad2*) subtypes (Figure 1, A and B, and Supplemental Figure 2C). Non-neuronal clusters included oligodendrocytes, immune cells, oligodendrocyte progenitor cells (OPCs), astrocytes, and vascular leptomeningeal cells (Figure 1A and Supplemental Figure 2C). Samples from the CPZ group exhibited oligodendrocyte loss alongside increased immune cell and OPC counts relative to baseline (Figure 1B); these findings were consistent with oligodendrocyte cell death, inflammation, and increased remyelination-mediating OPCs expected with CPZ exposure (15).

Because PEX5 loss was targeted to myeloid cells and microglia, we performed immune cell subanalysis. Among immune cells, we detected 9 subclusters, of which 7 comprised microglia, characterized by *Hexb* and *Tgfbr1* enrichment (Figure 1, C–E, and Supplemental Table 2). The remaining 2 nonmicroglial clusters included macrophages (expressing *Mrc1* and *F13a1*) and lymphocytes (expressing *Skap1* and *Itk*) (Figure 1E). Under baseline conditions, the bulk of microglia expressed the homeostatic genes *P2ry12* and *Cx3cr1* (16) (Figure 1, C, E, and F).

Within the CPZ arm, microglia underwent a marked shift in cell states. Microglia exhibiting intermediate expression of activation-related genes (*Axl*, *Apoe*) were labeled as intermediate microglia (Figure 1E). Microglia that strongly upregulated activation-related genes and downregulated homeostatic genes were classified as DMAMs and were subclassified into 3 clusters labeled DMAM0, DMAM1, and DMAM2 (Figure 1E). Relative to DMAM0, the DMAM1 and DMAM2 clusters acquired additional markers beyond hallmark activation genes. Among marker genes, DMAM1 exhibited high expression of *Lpl* and *Csf1* and intermediate expression of *Lgals3*, whereas DMAM2 demonstrated high expression of *Lgals3* and high expression of *Gpnmb* (Figure 1, C and E). Consistent with an inflammatory response, we also identified proliferating microglia (i.e., cycling) and interferon-responsive microglia enriched for interferon pathway genes (Figure 1E).

Notably, the DMAM2 cluster was almost exclusively identified in PEX5cKO samples within the CPZ arm, whereas control DMAMs predominantly consisted of DMAM0 and DMAM1 clusters (Figure 1F). Fold-change analysis confirmed a significant increase in the DMAM2 cell fraction in PEX5cKO CPZ samples, accompanied by a reduction in DMAM0 and DMAM1 cell fractions relative to control CPZ samples (Figure 1G).

We next performed pseudotime analysis to elucidate the developmental trajectory of the DMAM clusters (17). Selecting homeostatic microglia as the origin cluster, pseudotime analysis revealed a sequential developmental trajectory of homeostatic microglia into intermediate and DMAM0 microglia (Figure 1H). From the DMAM0 state, activated microglia then diverged into DMAM1 or DMAM2 states (Figure 1, H–J). Coupling pseudotime and fold-change analyses indicated that PEX5cKO reprogrammed the DMAM evolution, redirecting DMAMs away from the DMAM1 state and toward the PEX5cKO-enriched DMAM2 state.

### PEX5cKO undermines Apoe expression in activated microglia.

**Figure 2:**
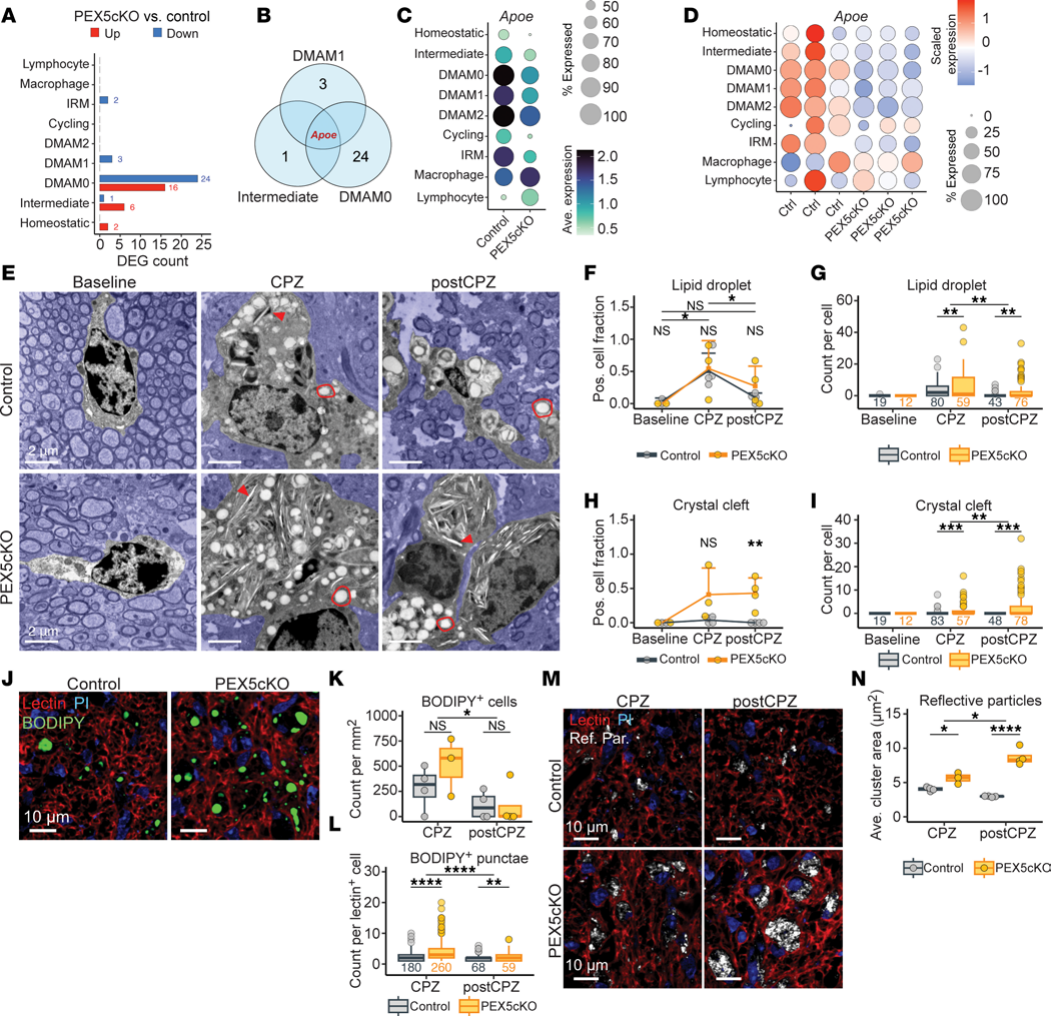
PEX5 loss impairs *Apoe* expression, aggravating lipid droplet burden and promoting lipid crystal accumulation. (**A**) Upregulated and downregulated pseudobulk-derived DEGs per immune subcluster. Numbers over bars indicate DEG count >0. (**B**) Venn diagram triangulating downregulated DEGs detected in DMAM0, DMAM1, and intermediate subclusters. (**C**) *Apoe* average expression per immune subcluster split by genotype. (**D**) Per immune subcluster *Apoe*-scaled expression across libraries within the CPZ condition. (**E**) Representative TEM micrographs of phagocytes. Red circles and red arrowheads mark lipid droplets and crystal clefts, respectively. Nonphagocyte area is shaded blue to ease visualization of phagocyte. Scale bar: 2.0 μm. (**F**) Lipid droplet–positive cells as fraction of total cells imaged. (**G**) Lipid droplet count per cell assessed. (**H**) Crystal-positive cells as a fraction of total cells imaged. (**I**) Crystal cleft count detected per cell. (**J**) Representative micrographs for BODIPY and lectin staining within the CPZ condition. Nuclei are stained with propidium iodide dye. Scale bar: 10 μm. (**K**) BODIPY–positive cell count /mm^2^. (**L**) BODIPY–positive droplet count per lectin–positive cell. (**M**) Representative micrographs of dual confocal and reflective microscopy imaging lectin stain and reflective particles (Ref. Par.). Nuclei are stained with propidium iodide. Scale bar: 10 μm. (**N**) Average area of reflective particle microclusters. (**E**–**N**) Representative images were acquired from corpus callosum; cells analyzed and quantitated were imaged from corpus callosum. (**F**, **H**, **K**, and **N**) Individual data points correspond to biological replicates. (**F** and **H**) Square and error bar correspond to mean and SD, respectively. (**G**, **I**, and **L**) Data points represent dataset outliers. Cell number assessed per group are given below each box plot. Statistical analysis for **F–I**, **K**, **L**, and **N**) involved ANOVA followed by Tukey’s HSD. Adjusted *P* values are indicated as follows: NS indicates *P* > 0.05; *P < 0.05, **P < 0.01, ***P < 0.001, ****P < 0.0001. IRM, interferon-responsive microglia; Pos, positive.

To identify molecular changes driving the altered DMAM states downstream of PEX5 depletion, we performed pseudobulk analysis comparing control and PEX5cKO genotypes across the immune clusters within the CPZ arm. Using an adjusted *P*-value cutoff of 0.15, we identified relatively few differentially expressed genes (DEGs) across the subclusters (Figure 2A and Supplemental Table 3). No DEGs were identified in the DMAM2 cluster, likely because the cluster predominantly comprised PEX5cKO cells (Figure 2A). However, triangulation of downregulated DEGs revealed that *Apoe* expression was decreased in PEX5cKO samples across the DMAM0, DMAM1, and intermediate clusters (Figure 2B). The *Apoe* gene encodes for apolipoprotein E (APOE), a critical activation-related protein. *Apoe* expression is strongly induced in DMAMs after demyelination, as was observed in our snRNA-Seq data (Figure 2C), which demonstrated both increased average as well as higher percent expression of *Apoe* in intermediate and DMAM clusters relative to homeostatic microglia. However, *Apoe* expression in the intermediate and DMAM clusters was significantly impaired in PEX5cKO samples (Figure 2D). Thus, *Apoe* expression in the context of CPZ-induced demyelination is dependent on PEX5 and, presumably, peroxisome integrity in microglia.

### Impaired Apoe expression correlates with exacerbated lipid droplet accumulation and crystal precipitation in PEX5cKO tissue.

After cell debris uptake, DMAMs upregulate lipid droplet biogenesis and *Apoe*-dependent lipid export (18). *Apoe* downregulation and impaired lipid export undermine the pro-remyelinating properties and exacerbate lipid droplet burden in microglia (19). To assess for lipid droplets, we used transmission electron microscopy (TEM) to quantitate lipid droplets within lesion-associated phagocytes. Additionally, to assess the impact of PEX5 depletion on remyelination, a third experimental arm was added, wherein after 6 weeks of a CPZ diet, PEX5cKO and control littermates were switched back to standard chow for 10 days (post-CPZ) (Supplemental Figure 1G).

Using TEM, lipid droplets were visualized in CPZ and post-CPZ conditions relative to baseline, consistent with the expected increase in lipid droplet biogenesis in DMAMs during demyelination (Figure 2, E–G). In agreement with impaired *Apoe* gene expression, we observed that lipid droplet counts per phagocyte were elevated in PEX5cKO relative to controls in both the CPZ and post-CPZ conditions (Figure 2, E–G). Lipid droplet burden peaked in the CPZ arm and declined in the post-CPZ arm for both PEX5cKO and controls, indicating that although PEX5 loss exacerbated lipid droplet burden, lipid droplet clearance remained intact (Figure 2, E–G).

Lipid droplet processing requires the export of cholesterol via lipoproteins, including APOE (20). Control DMAMs expressing APOE are expected to more effectively extrude lipids via lipoprotein-dependent mechanisms. *Apoe* downregulation in PEX5cKO DMAMs suggests the resolution of lipid droplets was associated with defective lipid export. Impaired lipid export after lipid droplet catabolism can lead to aberrant accumulation of lipids in other subcellular structures, including lipid crystals (19). Elevated intracellular sterol concentration secondary to APOE loss in myelin debris–clearing microglia is associated with sterol crystal precipitation (19). Accordingly, we observed a marked increase in crystal clefts within PEX5cKO phagocytes (Figure 2, H and I). Both frequency of crystal positive phagocytes and per-cell crystal burden peaked in PEX5cKO post-CPZ samples, following an inverse trend relative to lipid droplet burden (Figure 2, H and I). In contrast, crystals were less frequently observed in control samples during demyelination and remained undetected within control post-CPZ samples (Figure 2, H and I). Therefore, unlike in control samples, lipid droplet resolution in PEX5cKO samples was directly associated with crystal accumulation.

Consistent with the TEM analysis, lipid droplets were readily detected in lectin^+^ phagocytes after demyelination using BODIPY dye (Figure 2J). Although BODIPY^+^ cell counts resolved in the post-CPZ arm across genotypes (Figure 2K), BODIPY^+^ punctae remained increased in PEX5cKO lectin^+^ phagocytes relative to controls (Figure 2L), thus validating our TEM assessment that PEX5 loss exacerbated lipid droplet burden without blocking lipid droplet resolution. Crystals in tissue can be detected as reflective particles, using reflection microscopy. Accordingly, dual confocal and reflection microscopy detected microclusters of reflective particles within lectin^+^ phagocytes in both the CPZ and post-CPZ conditions (Figure 2, M and N). Importantly, the average area for reflective particle microclusters was significantly higher in PEX5cKO lectin^+^ phagocytes relative to controls and increased in the post-CPZ condition relative to the CPZ condition, suggestive of higher crystal burden in PEX5cKO post-CPZ samples after lipid droplet resolution (Figure 2, M and N). Thus, our tissue-staining, reflective microscopy, and electron microscopy data are consistent with impaired lipid processing in PEX5cKO DMAMs.

In summary, control samples exhibited an expected pattern of lipid droplet biogenesis and resolution within the CPZ and post-CPZ conditions. In contrast, PEX5cKO DMAMs exhibited an exacerbated burden of lipid droplets and accumulation of intracellular crystals correlating with lipid droplet resolution.

### Impaired lipophagy in PEX5cKO correlates with intralysosomal crystals and curvilinear profiles.

**Figure 3:**
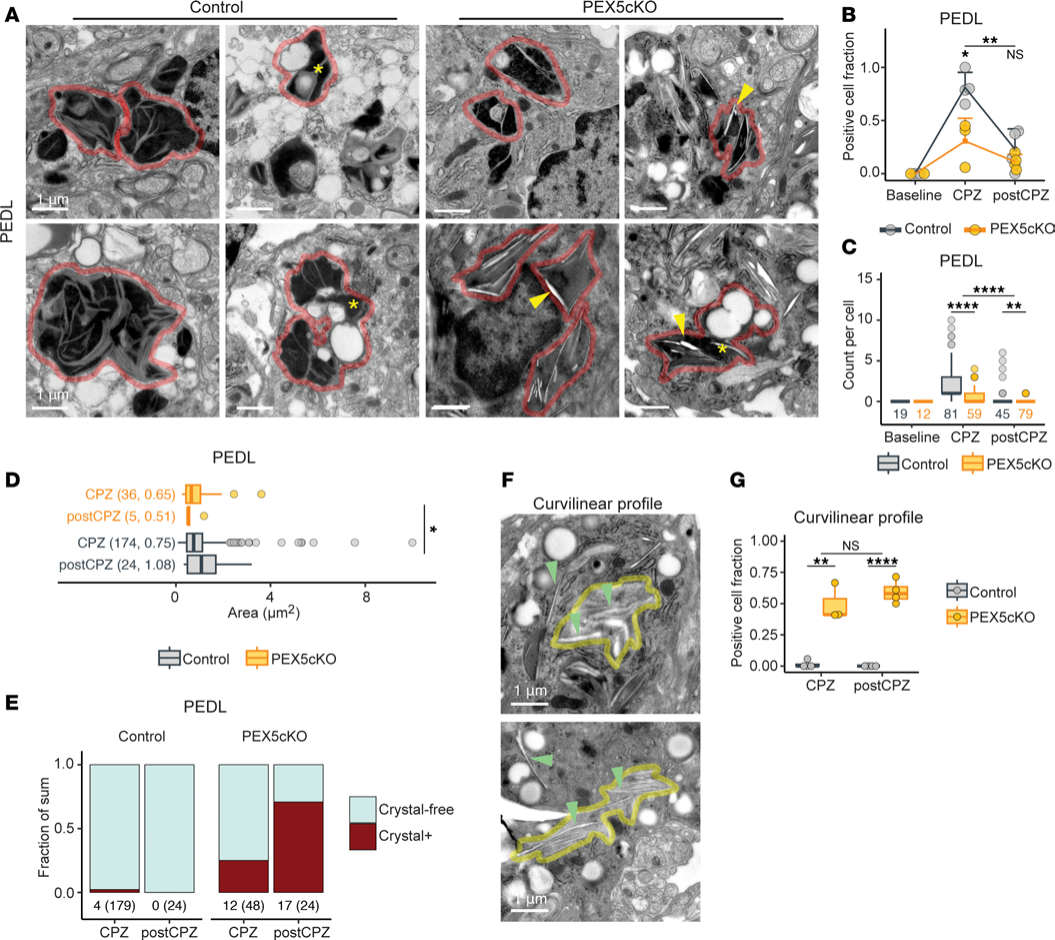
PEX5cKO phagocytes exhibit intralysosomal crystals and cytosolic curvilinear profiles. (**A**) Representative transmission electron micrographs of PEDLs indicated with a imaged phagocyte outlined in red. Yellow asterisks mark PEDLs fusing with lipid droplets, evocative of lipophagy. Yellow arrows indicate intralysosomal crystal clefts. Scale bar: 1.0 μm. (**B**) PEDL-positive cell quantification as fraction of total cells imaged. Individual data points correspond to biological replicates. Square and error bar correspond to mean and SD, respectively. (**C**) PEDL count per cell. Data points correspond to outliers and number of cells assessed are given below each box plot. (**D**) Area, in μm^2^, per crystal-free PEDL grouped by genotype and condition. PEDL count and median area are indicated in parentheses. Data points correspond to outliers. (**E**) Fraction of summed PEDLs corresponding to crystal-free and -positive PEDLs, grouped by genotype and condition. Crystal-positive PEDL counts are indicated below stacked bars. Crystal-free and -positive sums are indicated in parentheses. (**F**) Representative transmission electron micrographs of curvilinear profiles detected in phagocytes within corpus callosum after CPZ exposure. Green arrowheads indicate representative curvilinear profiles. Yellow border surrounds cluster of curvilinear profiles. Scale bars: 1.0 μm. (**G**) Curvilinear profile–positive cells as fraction of total cells imaged. Individual data points correspond to biological replicates. All representative images were acquired from corpus callosum. Quantifications only used cells imaged from corpus callosum. (**B**, **C**, and **G**) Statistical analysis for included ANOVA followed by Tukey’s HSD. Adjusted *P* values are indicated as follows: NS indicates *P* > 0.05; *P < 0.05, **P < 0.01, ***P < 0.001, ****<0.0001. (**D**) Unpaired, 2-tailed t test, *P < 0.05.

Lipid droplets are processed in part by lipophagy, which enables autophagy-mediated lipid catabolism through fusion of lipid droplets with lysosomes (21, 22). In control samples after CPZ-mediated demyelination, large (0.75 μm^2^ and 1.08 μm^2^ median areas in CPZ and post-CPZ arms, respectively), polymorphic, electron-dense lysosomes (PEDLs) were observed fusing with lipid droplets within phagocytes, which is suggestive of lipophagy (Figure 3A). The frequency and intracellular count of PEDLs peaked during demyelination and correlated with the peak lipid droplet density already described (Figure 3, B and C).

Interestingly, relative to controls, PEDLs were scarcer in PEX5cKO phagocytes (Figure 3, B and C) and had smaller areas (0.65 μm^2^ and 0.51 μm^2^ median areas in CPZ and post-CPZ, respectively) (Figure 3D). Moreover, PEX5cKO phagocytes had a higher incidence of crystal clefts within PEDLs (Figure 3E), suggestive of impaired lipid extraction from lysosomes, leading to lipid accumulation and crystal precipitation. Thus, loss of PEX5 and peroxisome integrity present with findings suggestive of impaired intracellular lipid processing after debris engulfment, including lipophagy disruption and accumulation of intralysosomal crystals.

In addition to intralysosomal crystal accumulation, we observed the formation of hypodense, curvilinear profiles within PEX5cKO phagocytes during and after CPZ exposure (Figure 3F), with greater than 50% of PEX5cKO phagocytes in the post-CPZ condition presenting with the same profiles (Figure 3G). Pathology reports described the occurrence of laminar profiles in brain phagocytes in cases with adrenoleukodystrophy that were hypothesized to consist of lipid deposits with very-long-chain fatty acids (23, 24). The enrichment of curvilinear profiles in PEX5cKO phagocytes therefore raises the possibility of a convergence in pathologies between brain phagocytes in CPZ-fed PEX5cKO mice and adrenoleukodystrophy.

### PEX5cKO DMAMs exhibit exacerbated GAL3 response and lysosome turnover.

**Figure 4:**
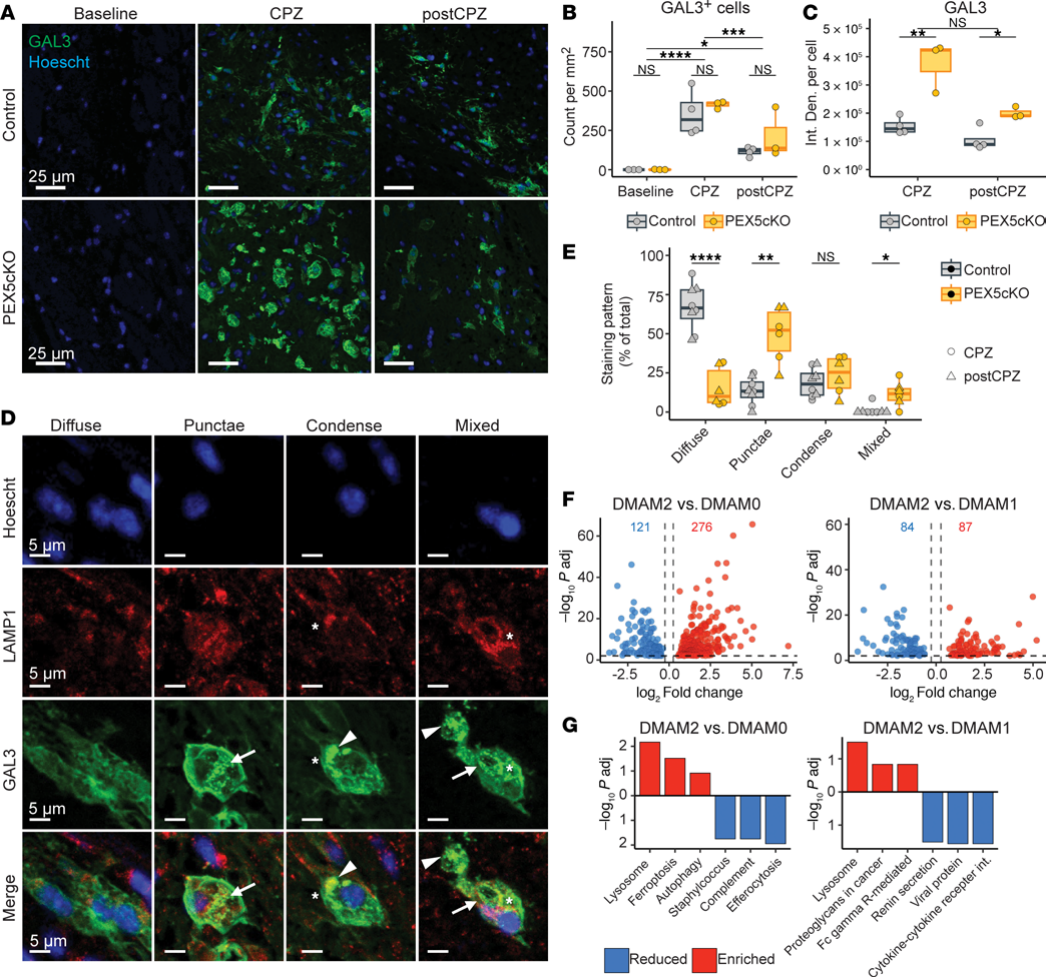
Exacerbated GAL3 response and lysosome turnover in PEX5cKO DMAMs. (**A**) Representative micrographs for GAL3 immunofluorescence within corpus callosum. Nuclei are stained with Hoescht dye. Scale bar: 25 μm. (**B**) GAL3^+^ cell count per mm^2^ of corpus callosum. Individual data points correspond to biological replicates. (**C**) Average GAL3 fluorescence integrated density (Int. Den.) per GAL3^+^ cell. Individual data points correspond to biological replicates. (**D**) Representative micrographs of GAL3^+^ cells with punctae (arrows), condensed (arrowheads), and mixed staining patterns, which frequently colocalize with LAMP1 staining (asterisk). Scale bar = 5 μm. (**E**) Percentage of GAL3^+^ cells subdivided by staining pattern represented in **D**. Individual data points correspond to biological replicates. Color corresponds to genotype, and data point shape corresponds to condition. (**F**) Upregulated (red) (–log_10_ adjusted *P* > 2.0; log_2_ fold change > 0.25) and downregulated (blue) (–log_10_ adjusted *P* > 2.0; log_2_ fold change < –0.25) DEGs detected in the DMAM2 subcluster relative to DMAM0 (left) and DMAM1 (right) subclusters, respectively. (**G**) Top 3 enriched and reduced KEGG pathways in DMAM2 relative to DMAM0 (left) and DMAM1 (right) subclusters, respectively. (**A–E**) Representative images were acquired from corpus callosum. Quantifications only used cells imaged from corpus callosum. (**B** and **C**) Statistical analysis included ANOVA followed by Tukey’s HSD. (**E**) FDR-adjusted, 2-tailed t test. Adjusted *P* values are indicated as follows: NS indicates *P* > 0.05; *P < 0.05, ** *P* < 0.01, *** *P* < 0.001, **** *P* < 0.0001. p.adj., adjusted *P* value.

Intralysosomal crystals can compromise lysosome integrity and induce cell stress (19). Accordingly, PEX5cKO samples had a significantly higher number of cycling microglia (Figure 1G), suggestive of higher microglial turnover secondary to cell death from lysosome injury. Additionally, snRNA-Seq revealed an increase in *Lgals3* expression in the DMAM2 state enriched in PEX5cKO samples (Figure 1E). *Lgals3* encodes for galectin 3 (GAL3), a glycoprotein-binding factor involved in lysosome damage response (25, 26). Whereas *Lgals3* was almost undetectable in homeostatic and intermediate microglial clusters, *Lgals3* achieved low expression in DMAM0 and interferon-responsive microglia clusters, and intermediate and high expression in DMAM1 and DMAM2 clusters, respectively (Figure 1E). The expression pattern for *Lgals3* is consistent with previous reports describing GAL3 upregulation in activated, but not resting, microglia (27). Consistent with the paucity of DMAMs at the baseline condition, immunofluorescence analysis demonstrated that GAL3^+^ cells were nearly undetectable in both control and PEX5cKO baseline samples (Figure 4, A and B). In the CPZ arm, GAL3^+^ cell counts significantly increased, reflecting the emergence of DMAMs during demyelination. In the post-CPZ arm, GAL3^+^ cell counts dropped, consistent with a resolution in demyelination (Figure 4, A and B). Overall, GAL3^+^ staining correlated with the expected dynamics for DMAM cell counts associated with CPZ use.

GAL3^+^ cell counts did not substantially differ between the PEX5cKO and control samples under CPZ and post-CPZ conditions (Figure 4B). However, in agreement with the transcriptomic analysis, PEX5 depletion increased GAL3 staining intensity per cell relative to controls after CPZ-mediated demyelination (Figure 4C). Thus, PEX5 KO promoted a shift favoring GAL3-high expressing DMAMs, congruent with the enrichment of *Lgals3*-high expressing DMAM2 cluster in PEX5cKO samples.

In addition to increased GAL3 expression, PEX5cKO DMAMs also exhibited greater formation of intracellular GAL3^+^ punctae that frequently colabeled with lysosomal-associated membrane protein 1 (LAMP1) (Figure 4, D and E). After lysosome injury, GAL3 localizes to the exposed lysosomal lumen, enabling a signaling cascade driving lysosome repair and turnover (28). GAL3 upregulation and robust formation of GAL3^+^ punctae in PEX5cKO DMAMs suggested an intact response to lysosome injury expected with intralysosomal crystals detected with TEM. Accordingly, using DEG and pathway enrichment analyses between clusters, we observed that relative to both DMAM1 and DMAM0, the DMAM2 cluster was significantly enriched for the lysosome gene pathway, which is compatible with elevated lysosome turnover secondary to injury and expected with an activated, GAL3-dependent injury response. (Figure 4, F and G, and Supplemental Table 4).

Thus, transcriptomic and imaging data indicate PEX5 KO and loss of peroxisome integrity undermine lysosome-mediated degradation of lipids traceable through intralysosomal crystals, GAL3 upregulation and redistribution, and increased lysosome turnover.

### Myelin-debris uptake in PEX5cKO macrophages can partially reproduce the DMAM2 cluster-associated signature.

Our data indicate that DMAMs responding to demyelination depend on PEX5 for mitigating crystal accumulation. A primary function of DMAMs is myelin-debris clearance. To assess whether perturbations in PEX5cKO DMAMs were unmasked by myelin-debris uptake, we asked whether challenging PEX5cKO macrophages with myelin debris could replicate DMAM2 cluster-associated signatures.

We generated bone marrow–derived macrophages from control and PEX5cKO littermates. In all conditions, culture medium was supplemented with 4-hydroxytamoxifen to enable PEX5 depletion. Macrophages were supplemented with purified myelin debris for 24 hours and 72 hours and collected for bulk RNA-Seq. Myelin debris–naive macrophages (resting) served as baseline references (Supplemental Figure 3A).

Principal component analysis (PCA) and DEG analysis demonstrated that PEX5 KO had a subdued impact under baseline conditions, with minimal separation between resting macrophage transcriptomes (Supplemental Figure 3B), consistent with our findings in vivo where PEX5cKO and control homeostatic microglia remained comparable. Next, after myelin-debris engulfment, macrophage transcriptomes diverged significantly between the PEX5cKO and control genotypes (Supplemental Figure 3B), with the number of DEGs increasing to 244 at 24 hours, and further increasing to 1,115 after 72 hours of myelin-debris exposure (Supplemental Figure 3C and D, and Supplemental Table 5). The higher DEG count in bone marrow–derived macrophages relative to DMAMs likely reflected a higher sensitivity to transcriptomic changes afforded by deeper bulk sequencing relative to snRNA-Seq and inherent differences between bone marrow–derived macrophages and microglia and between in vitro and in vivo conditions. However, 16 of 87 DEGs upregulated in the DMAM2 cluster relative to DMAM1 were also upregulated in PEX5cKO macrophages relative to control after 72 hours of myelin exposure (Supplemental Figure 3E). Importantly, the shared DEGs included *Gpnmb* and *Lgals3* (Supplemental Figure 3, E and F). Additionally, pathway enrichment analysis exhibited enrichment for lysosome pathways in PEX5cKO macrophages relative to controls across both time points of myelin-debris exposure, as had been seen with DMAM2 (Supplemental Figure 3G).

Interestingly, *Apoe* expression was not affected by PEX5 status in bone marrow–derived macrophages, either at baseline or following myelin-debris exposure, indicative that reliance of *Apoe* expression on PEX5 remains context or cell-type dependent (Supplemental Figure 3F). However, Ingenuity pathway upstream regulator activity analysis inferred impaired APOE activity at both time points of myelin-debris exposure (Supplemental Figure 3H), indicating that although *Apoe* expression remained intact in PEX5cKO macrophages, APOE-related pathways were deficient, including reduced expression of *Abca1* (Supplemental Figure 3F). Finally, gene set enrichment analysis of transcriptional signatures derived from published snRNA-Seq datasets demonstrated that foamy microglia signatures were enriched in PEX5cKO macrophages relative to control post–myelin debris uptake (Supplemental Figure 3I and Supplemental Table 6), consistent with the exacerbated lipid laden phenotype we observed in vivo in PEX5cKO DMAMs.

In summary, myelin-debris uptake in PEX5cKO bone marrow–derived macrophages can partially reproduce the molecular signature associated with the DMAM2 cluster, indicating that myelin debris uptake in PEX5cKO phagocytes is a contributing factor to DMAM2 cluster emergence.

### Emergence of the DMAM2 cluster in PEX5cKO samples correlates with impaired remyelination.

To assess the impact of DMAM disruption on remyelination, we analyzed the oligodendroglia lineage within the snRNA-Seq dataset. Our dataset included OPCs and 9 subclusters of *Mbp^+^* oligodendrocytes (Supplemental Figure 4, A and C, and Supplemental Table 7). Cell-marker annotation elucidated committed oligodendrocyte progenitors and newly formed oligodendrocytes notable for *Frm4da* and *Tcf7l2* enrichment (Supplemental Figure 4, A and C); myelin-forming oligodendrocytes (MFOLs), which upregulated *Man1a* and *Synpr* and myelin genes including *Mog* and *Mobp* (Supplemental Figure 4, A and C); and mature oligodendrocytes (MOL), which downregulated immature marker *Pcdh7* and upregulated the genes *Il33* and *Ptgds* associated with MOL subtype 5/6 (MOL5/6) or the gene *Anln* associated with MOL subtype 2 (MOL2) (Supplemental Figure 4, A and C). We also detected 2 additional variations of MOL5/6 notable for enrichment of *Adgrv1* (*Adgrv1*^+^ MOL5/6) and *Clca4a* (*Clca4a*^+^ MOL5/6).

In the CPZ arm, we observed the emergence of a demyelination-associated oligodendrocyte (DOL) cluster notable for enrichment for *Tenm4* expression and only weak expression of MOL5/6 and MOL2 markers (Supplemental Figure 4, A and C). Two additional clusters emerged—*Sox6*-high DOLs (*Sox6^+^* DOL) and interferon-responsive oligodendrocytes—with interferon pathway gene upregulation (Supplemental Figure 4, A and C). Interestingly, pseudotime analysis indicated that DOLs emerged from MFOLs in the context of CPZ exposure, suggesting that continual exposure to CPZ shifts MFOLs from the MOL lineage and toward the DOL state (Supplemental Figure 4F).

PEX5cKO status did not significantly affect cell cluster fractions when assessing fold-change relative to controls (Supplemental Figure 4, B, D, and E). Moreover, pseudobulk analysis between PEX5cKO and controls across clusters revealed relatively few DEGs (Supplemental Table 8). Interestingly, DOLs demonstrated a marked upregulation of *Apod* gene expression (Supplemental Figure 4, G–I), which is associated with oligodendrocyte stress response during ischemia (29, 30) and is suggestive of an elevated stress-promoting environment secondary to PEX5 KO in DMAMs.

**Figure 5:**
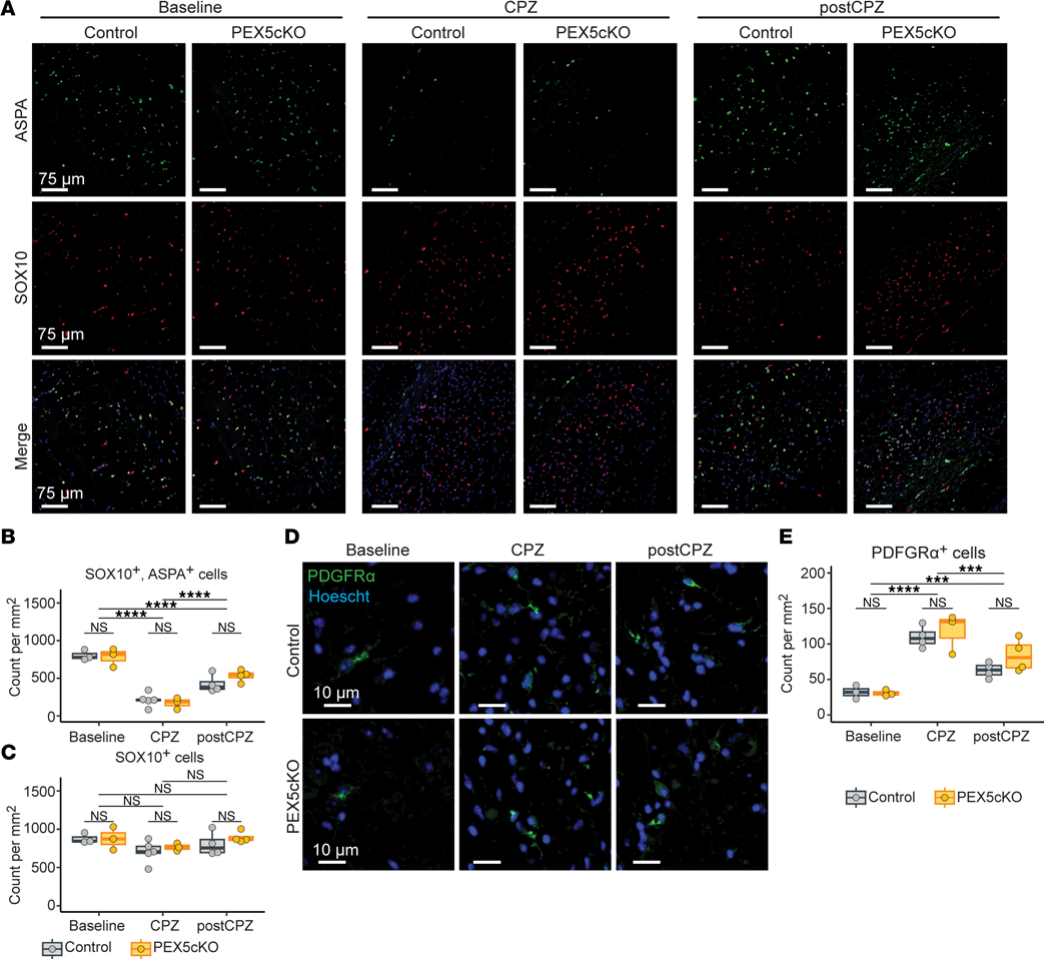
Oligodendrocyte density remains comparable between PEX5cKO and control genotypes across conditions. (**A**) Representative confocal micrographs for ASPA and SOX10 immunofluorescence. Nuclei are stained with Hoescht dye. Scale bar: 75 μm. (**B**) SOX10^+^ cell count per mm^2^ in corpus callosum. (**C**) ASPA^+^, SOX10^+^ cell count per mm^2^ in corpus callosum. (**D**) Representative confocal micrographs for PDGFRα^+^ immunofluorescence. Nuclei are stained with Hoescht dye. Scale bar: 10 μm. (**E**) PDGFRα^+^ cell count per mm^2^ in corpus callosum. **A–E**) Representative images were acquired from corpus callosum. Quantifications only used cells imaged from corpus callosum. Individual data points per box plot correspond to biological replicates. (**B**, **C**, and **E**) Statistical analysis for included ANOVA followed by Tukey’s HSD, Adjusted *P* values are indicated as follows: NS indicates *P* > 0.05; ***P < 0.001, ****P < 0.0001.

**Figure 6:**
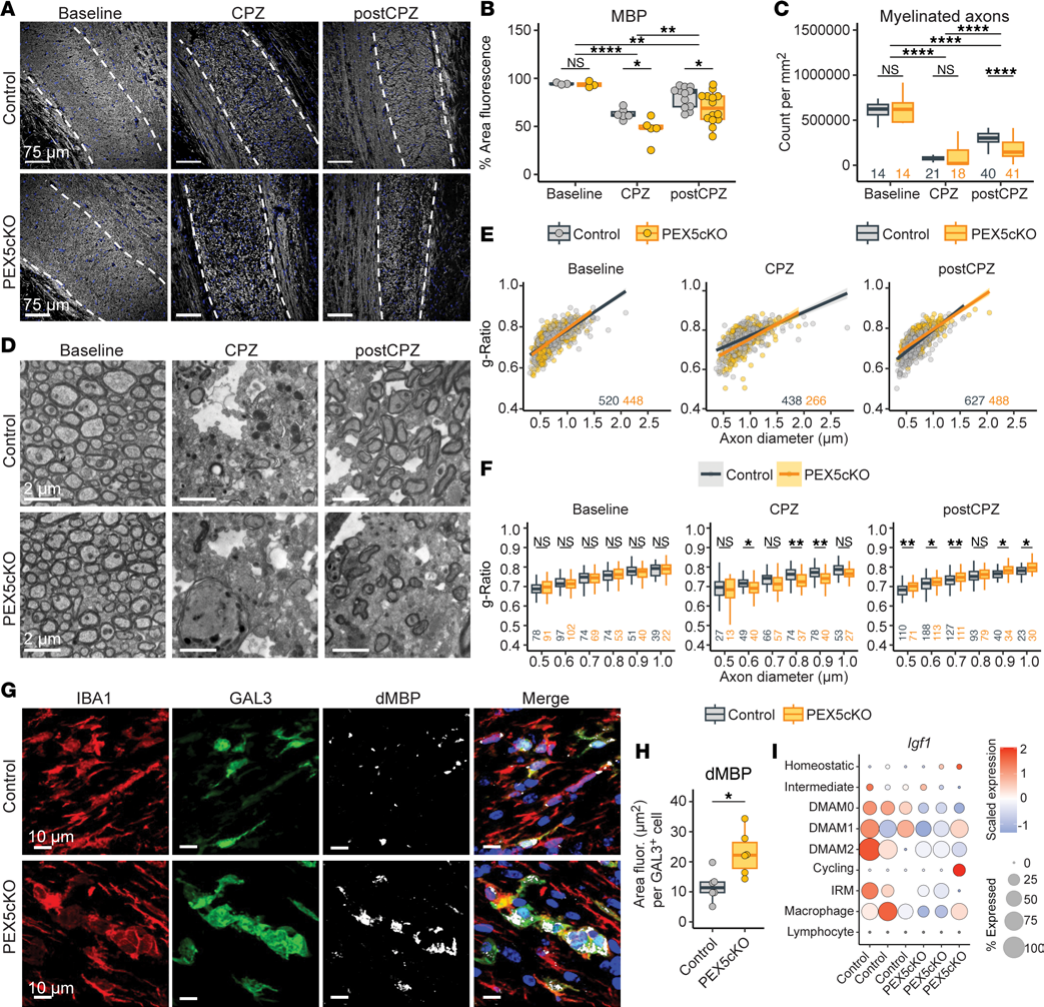
PEX5 deficiency in microglia impairs myelin-debris clearance and remyelination. (**A**) Representative confocal micrographs for MBP immunofluorescence. Nuclei are stained with Hoescht dye. White dotted lines correspond to corpus callosum borders. Scale bars: 75 μm. (**B**) MBP^+^ percent area fluorescence. Individual data points correspond to biological replicates. (**C**) Myelinated axon count per mm^2^ of tissue. Number of micrographs analyzed are given below each box plot. (**D**) Representative transmission electron micrographs of myelinated axons. Scale bar: 2.0 μm. (**E**) Myelinated axon g-ratio distribution across axon diameters. Lines correspond to linear regression. Number of myelinated axons analyzed are given and colored according to genotype. (**F**) Myelinated axon g-ratio binned across axon diameters rounded to the nearest 0.1 decimal. Number of myelinated axons analyzed are given below each box plot. (**G**) Representative confocal micrographs for IBA1, GAL3, and dMBP immunofluorescence within the corpus callosum. Nuclei are stained with Hoescht dye. Scale bar: 10 μm. (**H**) dMBP^+^ area fluorescence (fluor.) per IBA1^+^, GAL3^+^ cell assessed. Data points correspond to biological replicates. (**I**) Average gene expression of *Igf1* scaled across biological replicates per immune subcluster detected within the CPZ condition. Dot color and size correspond to scaled gene expression and percentage expressed, respectively. (**B** and **C**) Statistical analysis included ANOVA followed by Tukey’s HSD comparison. (**F**) FDR-adjusted, 2-tailed *t* test. Adjusted *P* values are indicated as follows: NS indicates *P* > 0.05; *P < 0.05, **P < 0.01, ***P < 0.001, ****P < 0.0001. (**H**) Unpaired, 2-tailed *t* test, *P < 0.05. (**A–H**) Representative images were acquired from corpus callosum. Quantifications only used cells imaged from corpus callosum. (**C**, **E**, and **F**) Mice used per genotype for baseline, CPZ, and post-CPZ conditions were 2, 3, and 4, respectively.

Immunofluorescence analysis demonstrated that SOX10^+^, ASPA^+^ oligodendrocyte cell counts and PDGFRα^+^ OPCs remained comparable between PEX5cKO and controls across the baseline, CPZ, and post-CPZ conditions (Figure 5). However, we detected reduced myelin basic protein (MBP) staining in PEX5cKO samples relative to controls (Figure 6, A and B) in the CPZ and post-CPZ conditions. The MBP deficit between PEX5cKO and controls indicated a disruption in remyelination, which begins by 6 weeks of CPZ-diet consumption (15). TEM analysis validated a reduction in myelinated axon counts in PEX5cKO relative to the control, most prominently in the post-CPZ arm (Figure 6, C and D). Likewise, within the post-CPZ condition, we observed an elevated myelin g-ratio in PEX5cKO samples relative to controls, which is consistent with impaired remyelination (Figure 6, E and F). Furthermore, we observed an aggravated burden of injured axons detectable as deposits of synaptophysin in the corpus callosum of PEX5cKO animals relative to controls during remyelination, which is indicative of a higher incidence of axon damage associated with impaired remyelination (Supplemental Figure 4, J and K) (31).

Impaired myelin-debris clearance or loss of pro-remyelination factors provided by microglia can undermine remyelination (8). Accordingly, within DMAMs, we observed a marked accumulation of degraded myelin basic protein (dMBP), a marker for myelin debris, suggestive of a disruption in myelin-debris clearance (Figure 6, G and H). We also observed a decrease in pro-remyelinating factor *Igf1* expression in PEX5cKO DMAMs relative to controls (Figure 6I).

In summary, we report that loss of PEX5 and peroxisome integrity in DMAMs profoundly disrupts DMAM homeostasis and has adverse consequences reflected in impaired remyelination driven in part by loss of pro-remyelinating factors and impaired myelin-debris clearance.

## Discussion

Peroxisomes facilitate immunity-related tasks, including interferon expression (32), cytokine release, and pathogen clearance in macrophages (2–4). The present study demonstrates that peroxisomes also harbor a cell-intrinsic role in microglia critical to remyelination. Key findings indicate that impaired peroxisome biogenesis redirected the microglial response during demyelination, expanding a pool of crystal-laden DMAMs deficient in debris clearance and associated with impaired remyelination, lysosome injury markers, and downregulation of the critical sterol-exporter gene *Apoe*.

Using snRNA-Seq, marker gene expression coupled with pseudotime analysis revealed that CPZ-mediated demyelination resulted in robust recruitment of DMAMs, a subset of which acquired a lipid-laden phenotype, as indicated by lipid staining and TEM. PEX5 KO did not abate the formation of DMAMs but rather expanded an alternative DMAM state notable for lysosome gene set enrichment, lysosome damage–related genes *Lgals3* and *Gpnmb*, and exacerbated lipid droplet and crystal burden. *Gpnmb* and *Lgals3* expression is associated with lysosome stress and damage, including within microglia (33, 34). Researchers have associated foamy microglia enriched with GPNMB (a gene product of the *Gpnmb* human ortholog) with poorer outcomes in patients with multiple sclerosis, implicating GPNMB as a potential marker for lesion-exacerbating foamy microglia in multiple sclerosis (34). Conversely, the *Lgals3* gene product, GAL3, is a lysosome-damage response protein that localizes to the exposed lumen of injured lysosomes to help mediate lysosome repair and turnover (25, 26). Our findings align with those reports, revealing that *Lgals3* and *Gpnmb* expression correlates with lipid-laden microglia that exhibit features of lysosome stress, namely intralysosomal crystals and increased lysosome turnover, and with defective remyelination and exacerbated axonal injury. Importantly, our findings indicate that a pathomechanism leading to lysosome-stressed foamy microglia includes defective peroxisome biogenesis.

How disruption of peroxisome biogenesis contributes to lipid crystal accumulation remains undefined; however, *Apoe* gene downregulation suggests impaired sterol export could contribute to lipid saturation and crystal precipitation in PEX5cKO DMAMs. *Apoe* encodes for APOE, which is robustly induced in activated microglia (35). APOE KO mice have frequently been used as a model for atherosclerosis, giving rise to foamy cells laden with cholesterol crystals (19, 36). Importantly, APOE loss in microglia exacerbates cholesterol crystal precipitation during demyelination and impairs remyelination (19). Sterol export pathway gene products, including APOE, are transcriptionally regulated in part by liver X receptor (LXR), a nuclear receptor activated by oxysterols and sterol intermediates (37). During demyelination, DMAMs repurpose de novo sterol synthesis to upregulate desmosterol, which activates LXR and upregulates the sterol exporter genes *Abca1* and *Apoe*, facilitating lipid export and protecting against crystal precipitation (38). PEX5 regulates peroxisome biogenesis through post-translational mechanisms, mainly by translocating matrix proteins into peroxisomes (39), making direct transcriptional regulation a less plausible mechanism by which PEX5 could affect *Apoe* expression. However, peroxisomes have long been associated with sterol synthesis and are reported to house various sterol-synthesizing enzymes, including enzymes upstream of desmosterol (40, 41). Therefore, PEX5 KO could indirectly affect *Apoe* expression by undermining peroxisome-dependent synthesis of LXR-activating sterol intermediates. However, the extent and context of peroxisome contribution to sterol synthesis remains debated (42, 43).

An alternative mechanism relating impaired peroxisome integrity to crystal accumulation stems from our TEM findings. In contrast to PEX5cKO phagocytes, PEX5-intact phagocytes harbored large (frequently >1.0 μm in diameter) PEDLs fused with lipid droplets, structures evocative of lipophagy (21, 22). Moreover, the prevalence of PEDLs correlated with lipid-laden DMAMs during peak demyelination and resolved with lipid droplet clearance after CPZ removal. PEX5cKO DMAMs had fewer and smaller PEDLs, a higher fraction of which were crystal laden. Moreover, the lower number of PEDLs in PEX5cKO DMAMs correlated with a higher density of lipid droplets, suggesting an impaired lipophagy, a critical process in lipid droplet clearance that crosstalks with lipolysis (21, 22). Peroxisomes are reported to facilitate intracellular cholesterol trafficking between lysosomes and extra-lysosomal compartments (44). Loss of peroxisome biogenesis factors disrupted sterol export, leading to intracellular sterol accumulation (44). Plausibly, PEX5 depletion in DMAMs undermines the ability of peroxisomes to extract sterols from lysosomes, thus contributing to intra-lysosomal sterol saturation and crystal formation (45). Conversely, peroxisomes can integrate into multi-organelle units in activated macrophages, wherein peroxisomes facilitated the lipolysis of lipid droplets and release of inflammatory fatty acids in a PEX5-dependent manner (5). Interestingly, PEX5 has been implicated in starvation-related lipolysis by facilitating the translocation of adipose triglyceride lipase to lipid droplets (46). Therefore, peroxisome integrity may affect lipophagy by regulating lipid trafficking from lysosomes or through lipophagy-lipolysis crosstalk (22).

Myelin debris can impede remyelination by preventing maturation of newly formed oligodendrocytes (47, 48). Moreover, maturation of remyelinating oligodendrocytes relies on pro-remyelinating factors produced by DMAMs, including insulin-like growth factor (encoded by *Igf1*) (8, 49). Our data revealed both a deficit in myelin-debris clearance as well as reduced expression of the *Igf1* gene, indicating that PEX5cKO DMAMs were less efficient in providing a pro-remyelinating environment relative to their PEX5-intact counterparts. In macrophages, peroxisomes enable uptake and clearance of pathogens (3, 50). Interestingly, PEX5cKO DMAMs accumulated myelin debris relative to the control, suggesting a delay in myelin-debris degradation after internalization. Lipid dysregulation and accumulation disrupt lysosome function (51, 52), and lysosome dysfunction, in turn, impairs myelin debris clearance (53, 54). Therefore, defective lipid droplet clearance and elevated lipid and crystal burden in lysosomes could adversely interact with myelin-debris degradation within lysosomes, although it remains plausible that peroxisomes affect degradation of endolysosomal cargo independent of lipid droplet clearing mechanisms, including via generation of radical nitrogen and oxygen species (3).

Our data indicate peroxisomes are integral to DMAMs and may offer insights to peroxisomal disorders characterized by disruptions in microglia. Microglial dysfunction features prominently in adrenoleukodystrophy, a peroxisomal disorder driven by mutations to ATP-binding cassette subfamily D member 1 (encoded by *ABCD1*) and disruptions to very-long-chain fatty acid oxidation (9, 10, 55, 56). *Abcd1* KO mice challenged with CPZ and with MOG immunization exhibited altered neuroinflammatory responses that emulated adrenoleukodystrophy-related features, including blood-brain barrier disruption and perivascular infiltrates, indicating that peroxisome dysmetabolism perturbs neuroinflammatory responses (57). Our findings further indicate that cell-intrinsic disruptions of peroxisomal integrity in microglia are sufficient to undermine responses to demyelination, reinforcing a critical role for microglial peroxisome metabolism in peroxisomal disorders such as adrenoleukodystrophy. However, it should be emphasized that the generalizability of our findings targeting PEX5, which undermines peroxisome biogenesis and integrity, may be limited in diseases with intact peroxisome biogenesis, including adrenoleukodystrophy, in which PEX5 expression and function are conserved and in which pathogenic mechanisms stem from disruptions in specific peroxisomal pathways rather than global peroxisome dysfunction.

In summary, we report that myelin-debris clearance, sterol export, and lipid droplet metabolism in microglia depend on PEX5, presumably through peroxisome-related mechanisms. Our findings implicate an interrelationship between microglial peroxisomes and remyelination and encourage further exploration into the mechanisms interrelating peroxisomes and remyelination promoting properties in microglia.

## Methods

### Sex as a biological variable.

Both male and female animals were used, with similar findings across both sexes.

### Mouse strains and alleles.

Offspring heterozygous for the *Cx3cr1*^CreERT2-EYFP^ allele and homozygous for the *Pex5*^lox^ allele were used for conditional KO of PEX5 in myeloid cells, including microglia (*Cx3cr1*^CreERT2/wt^, *Pex5*^lox/lox^). Littermates heterozygous for *Cx3cr1*^CreERT2-EYFP^ and heterozygous or noncarrier for *Pex5*^lox^ (*Cx3cr1*^CreERT2/wt^, *Pex5*^lox/wt,wt/wt^) served as controls. To activate Cre-mediated recombination, PEX5cKO and control mice, aged 8–12 weeks, received daily 2 mg intraperitoneal injections of tamoxifen, dissolved in corn oil, for 5 consecutive days.

*Cx3cr1*^CreERT2-EYFP^ mice (catalog 021160) and *Pex5*^lox^ mice (catalog 031666) were obtained from The Jackson Laboratory and maintained under pathogen-free conditions at the MD Anderson Cancer Center Animal Facility.

### CPZ diet.

A 0.2% CPZ pellet diet (Envigo) was administered ad libitum to experimental mice for 6 weeks. Tamoxifen was administered 2 weeks prior to starting CPZ to enable turnover between matrix protein-intact and -deficient peroxisomes in PEX5 depleted myeloid cells.

### Bone marrow–derived macrophages.

Bone marrow isolate was extracted from femur and tibia using high g-force centrifugation (>10,000*g*, 30 seconds, 4°C) and collected in a sterile 1.5 mL microcentrifuge tube. Isolate was incubated in RBC lysis buffer (BioLegend) on ice for 5 minutes, followed by quenching with 10 volumes of ice-cold culture medium. Isolate was filtered through a 70 μm strainer, centrifuged at 500*g* for 10 minutes at 4°C, resuspended in culture medium, and trypan blue–stained live cells were counted. Isolate was seeded at a concentration of 1.0 × 10^5^ to 2.0 × 10^5^ live cells per milliliter in culture medium and cultured for 7 days for macrophage differentiation. Culture medium was replaced with fresh medium every 2–3 days, and consisted of 75% volume of l-glutamine–enriched RPMI 1640 medium, 10% FBS, and 15% L929 conditioned medium, with 100 U/mL penicillin-streptomycin. To deplete PEX5 in vitro, medium was supplemented with 1.0 μM 4-hydroxytamoxifen (MilliporeSigma, H7904) throughout the 7-day differentiation and during experimental assays for both PEX5cKO and control macrophages. The 4-hydroxytamoxifen stock solution was dissolved in 100% ethanol to 15 mM and added directly to culture medium at 1:15,000 dilution (final concentration 1.0 μM). Fresh 4-hydroxytamoxifen was added when replacing culture medium every 2–3 days.

### L929 conditioned medium.

L929 cells (mouse fibroblast cell line, ATCC CCL-1) with low passage number (<10) were plated in T175 flasks at a split ratio of 1:10 in DMEM with 10% FBS and 100 U/mL penicillin-streptomycin. After 8 days, conditioned medium was harvested and sterile filtered using a 0.22 μm filter and stored at –80°C. After thawing, conditioned medium was immediately used or stored in 4°C for no longer than 2 weeks.

### Immunofluorescence.

For formalin-fixed formaldehyde-embedded sections, anesthetized mice were transcardially perfused with 40 mL of 4.0% paraformaldehyde in PBS. Mouse brain was postfixed in 10% buffered formalin for minimum 48 hours at room temperature on a rocker. Formalin-fixed brains were dehydrated and embedded in paraffin and sectioned at 5 μm thickness onto pretreated glass slides. Sections were rehydrated and subjected to heat-mediated antigen retrieval in acidic citrate buffer with 0.05% Tween 20. Sections were blocked with blocking buffer (3% BSA, 1% horse serum in TBS with 0.1% Tween 20 [TBST]) for 1 hour and incubated with primary antibodies in blocking buffer overnight at 4°C. After washing in TBS, sections were incubated with fluorophore-conjugated secondary antibodies diluted 1:1,000 in blocking buffer for 1 hour at room temperature and protected from light. Slides were incubated with Hoescht 33342 (Invitrogen, Thermo Fisher Scientific, H1399) in TBS for 10 minutes and then washed in TBS. Coverslips (no. 1.5) were mounted with glycerol-based, antifade mounting solution (Invitrogen, Thermo Fisher Scientific, 36980) and cured at room temperature while protected from light.

For frozen sections, anesthetized mice were transcardially perfused with 40 mL of 4.0% paraformaldehyde in PBS. Mouse brain was postfixed in ice cold 4.0% paraformaldehyde in PBS overnight (~12 hours) at 4°C on a rocker. Fixed brains were cryoprotected by incubating in hyperosmolar sucrose solution (30% sucrose in PBS) at 4°C for 2–3 days. Brains were embedded in OCT compound and stored at –80°C. OCT-embedded brain tissue was cryosectioned into 7 μm thick sections placed on pretreated glass slides. Sections were rinsed with TBS and permeabilized using 1.0% Triton X-100 in TBS overnight at 4°C. After permeabilization, sections were blocked with blocking buffer (3% BSA, 1% horse serum in TBST) for 1 hour and incubated with primary antibodies in blocking buffer overnight at 4°C. After washing in TBS, sections were incubated with fluorophore-conjugated secondary antibodies diluted 1:1,000 in blocking buffer for 1 hour at room temperature and protected from light. Slides were incubated with Hoescht 33342 (Invitrogen, Thermo Fisher Scientific, H1399) in TBS for 10 minutes and then washed in TBS. Coverslips (No. 1.5) were mounted with glycerol-based, antifade mounting solution (Invitrogen, Thermo Fisher Scientific, 36980) and cured at room temperature while protected from light.

Primary antibodies included SOX10 (goat; R&D Systems, AF2864), ASPA (rabbit; EMD Millipore, ABN1698), MBP (mouse; BioLegend, 808401), GAL3 (rat; BioLegend, 125402), LAMP1 (rabbit; Abcam, ab24170), PDGFRα (rabbit; R&D Systems, AF1062), synaptophysin (rabbit; Abcam, ab32127), IBA1 (rabbit, Fujifilm Wako, 019-19741; goat, Fujifilm Wako, 011-27991), and dMBP (rabbit; MilliporeSigma, AB5864).

### Immunocytochemistry.

Bone marrow isolate was cultured on glass coverslips within 6-well plates. After macrophage differentiation, coverslips were washed with Dulbecco’s PBS (DPBS) and immediately fixed using 4.0% paraformaldehyde for 10 minutes at room temperature, followed by a second DPBS wash. For permeabilization, coverslips were incubated with 0.1% Triton-X100 in DPBS for 15 minutes at room temperature, followed by a DPBS wash. Coverslips were blocked with blocking buffer (3% BSA, 1% horse serum in TBST) for 1 hour and incubated with primary antibodies in blocking buffer overnight at 4°C. After washing in TBS, coverslips were incubated with fluorophore-conjugated secondary antibodies diluted 1:1,000 in blocking buffer for 1 hour at room temperature while protected from light, followed by incubation with Hoescht 33342 (Invitrogen, Thermo Fisher Scientific, H1399) in TBS for 10 minutes and a final wash in TBS. Coverslips were inverted onto pretreated slides with glycerol-based, antifade mounting solution (Invitrogen, Thermo Fisher Scientific, 36980) and cured at room temperature while protected from light.

### Lipid staining.

Whole brains were fixed, cryoprotected, OCT-embedded, and frozen as described previously under Immunofluorescence. After cryosectioning, 7 μm thick sections were incubated in open air at room temperature for 30 minutes, followed by 3 TBS washes of 10 minutes each. Cryosections were then incubated with 1.0 μM BODIPY 493/503 (Invitrogen, D3922), 5.0 μg/mL DyLight649-conjugated Griffonia Simplicifolia Lectin I, Isolectin B4 (Vector Laboratories, DL-1208), and propidium iodide (1:1,000) in TBS for 30 minutes at room temperature and protected from light. After washing sections with TBS, no. 1.5 coverslips were mounted using glycerol-based, antifade mounting solution (Invitrogen, 36980). Mounted slides were cured at room temperature, protected from light, and stored at –20°C.

### Immunoblotting.

Plated bone marrow–derived macrophages were washed twice with sterile DPBS and directly lysed using ice-cold RIPA lysis buffer (Thermo Fisher Scientific, 89900) supplemented with protease inhibitor cocktail (Roche, 11836170001; 1 tablet per 10 mL of RIPA) and phosphatase inhibitor solution (MilliporeSigma, P5726; 1:100 dilution in RIPA). After sonication and centrifugation at >10,000*g* for 15 minutes at 4°C, cleared supernatant was stored at –80°C. LDS sample buffer (Invitrogen, Thermo Fisher Scientific, NP0007) was added to 1× concentration and DTT (Roche 10197777001) was added to 50 mM concentration. Samples were heated to 95°C for 5 minutes and loaded onto precast tris-bis polyacrylamide gels for electrophoresis. Proteins were transferred onto nitrocellulose membranes via rapid semi-dry protein transfer. Membrane was blocked in 5% nonfat milk in TBST for 1 hour at room temperature. Blots were incubated with primary antibodies diluted 1:500–1,000 in TBST with 3% BSA overnight at 4°C. After washing with TBST, blots were incubated with HRP-conjugated secondary antibodies in TBST with 5% nonfat milk for 1 hour at room temperature and followed with a second wash in TBST. Blots were incubated with chemiluminescent substrate (Thermo Fisher Scientific, 34577), and images were acquired using x-ray films. Primary antibodies included PEX5 (rabbit; Proteintech, 12545-1-1AP) and β-actin (mouse; MilliporeSigma).

### RNA purification from bone marrow–derived macrophages.

Plated bone marrow–derived macrophages were washed with sterile DPBS twice and directly lysed with RLT lysis buffer (Qiagen, 79216) supplemented with 2-mercaptoethanol (1:100 dilution). Lysate was stored at –80°C until downstream RNA extraction. For RNA extraction, frozen lysate was thawed and then homogenized using QIAshredder columns (Qiagen, 79656) per the manufacturer’s instructions. After homogenization, RNA was purified using the Qiagen RNeasy mini columns (Qiagen, 74106) with DNA on-column digestion included using RNase-free DNase I (Qiagen, 79254), per the manufacturer’s instructions. Total RNA was eluted in nuclease free water and stored at –80°C.

### Real-time qPCR for bone marrow–derived macrophages.

Purified total RNA was reverse transcribed into cDNA using iScript reverse transcriptase (Bio-Rad, 1708841) per manufacturer’s instructions. Real-time qPCR was performed using the iTaq polymerase, universal SYBR green system (Bio-Rad, 1708841) on an Applied Biosystems 7500 Real-Time PCR system, per the manufacturer’s instructions.

### Bulk RNA-Seq for bone marrow–derived macrophages.

Purified total RNA with an RNA integrity number above 7.0 was submitted to Novogene for library preparation and next-generation sequencing. mRNA was enriched and cDNA libraries were generated from total RNA using the NEBNext Ultra II RNA library prep kit (New England Biolabs) per manufacturer’s instructions. cDNA libraries were pooled and sequenced on the NovaSeq 6000 to attain 20 × 10^6^ paired reads of 150 bases per read (20 million reads, paired-end sequencing 150 bp, 6 Gb) . Raw reads were recorded into FASTQ files and filtered for clean reads by removing (a) adapter-containing reads, (b) low-quality reads having more than 50% bases with a Q score of not greater than 5, and (c) reads composed of greater than 10% undetermined bases.

### Bulk RNA-Seq data processing and analysis.

Paired reads were aligned to the GRCm38 reference genome using graph-based alignment (HISAT2, version 2.1.0) (58). Aligned sequences were assembled into transcripts using the StringTie algorithm (version 2.2.1) (59). Coverage values generated per transcript were converted into hypothetical gene counts using the following formula: gene counts = coverage × (transcript length/read length). Raw gene counts were transformed using variance stabilizing transformation followed by dimensionality reduction using PCA to assess for batch effects and visualize patterns in low dimensional space. Raw gene counts were then inputted into the DESeq pipeline for normalization and DEG analysis (DESeq2, version 1.46.0) (60).

### Myelin-debris isolation and challenge.

Whole brains were excised from adult WT C57BL6 male mice and minced with a scalpel in ice-cold 0.32 M sucrose buffer (0.32 M sucrose, 20 mM Tris-HCl, 2 mM Na_2_EDTA, pH 7.45) supplemented with protease inhibitors (Roche, 11836170001). Minced brains were homogenized in 0.32 M sucrose buffer using a precooled Tenbroeck tissue grinder (Pyrex, 7727-07) on ice until achieving a smooth, milky homogenate. Homogenate was divided among 6 ultracentrifuge tubes, wherein 13 mL of homogenate was gently layered onto 17 mL of ice-cold 0.83 M sucrose buffer (0.83 M sucrose, 20 mM Tris-HCl, 2 mM Na_2_EDTA, pH 7.45) per tube, with care taken to avoid mixing between layers. The sucrose gradient was centrifuged at 75,000*g* for 1.5 hours at 4°C. The resulting intermediate layer was recovered and rehomogenized using 10 strokes in a precooled Tenbroeck tissue grinder on ice. Crude myelin was diluted in ice-cold hypotonic Tris-HCl buffer (20 mM Tris-HCl, 2 mM Na_2_EDTA, pH 7.45) and centrifuged at 33,000*g* for 15 minutes at 4°C. After decanting the supernatant, crude myelin was resuspended in Tris-HCl buffer and pelleted at 33,000*g* for 15 minutes at 4°C. To enhance myelin purity, crude myelin was resuspended in 0.32 M sucrose buffer and submitted to a second sucrose gradient centrifugation followed by 2 repeated osmotic shocks in hypotonic Tris-HCl buffer as described above. After the last osmotic-shock step, myelin debris was resuspended in ice-cold sterile DPBS and transferred to preweighed 1.5 mL microtubes. Myelin debris was pelleted at 22,000*g* for 10 minutes at 4°C and weighed, and then resuspended in sterile DPBS to a stock concentration of 200 mg/mL and stored at –80°C. We used 10 to 12 adult mice per batch of purified myelin. For myelin-debris challenge, myelin debris was thawed and homogenized by passing 5 times through a sterile 30G needle. Myelin debris was added to the culture medium to a concentration of 0.50 mg/mL for durations indicated in the study.

### Transmission electron microscopy.

Anesthetized mice were transcardially perfused with 40 mL of 4.0% paraformaldehyde in PBS. Mouse brains were hemisected and incubated in EM fixative solution (2.5% EM-grade glutaraldehyde and 2% EM-grade paraformaldehyde in 0.1 M cacodylate solution buffered to 7.4 pH) at 4°C for a minimum of 3 days on a rocker. A coronal section 1 mm thick was then taken from the coronal plane containing dorsal hippocampus (bregma –1.2 mm to –2.2 mm), the plane of which includes the corpus callosum area most affected by the 0.2% CPZ diet. Tissue was resected to achieve a section area of 1.0 mm^2^ and submitted to the High Resolution Electron Microscopy Facility at MD Anderson for processing and imaging. Samples were washed in 0.1 M sodium cacodylate buffer, treated with 0.1% Millipore-filtered cacodylate buffered tannic acid, postfixed with 1% buffered osmium tetroxide, and stained en bloc with 1% Millipore-filtered uranyl acetate. Samples were dehydrated in increasing concentrations of ethanol, infiltrated, and embedded in LX-112 medium. Samples were polymerized in a 60°C oven for approximately 3 days. Ultrathin sections were cut in a Leica Ultracut microtome, placed on formvar-coated, single-slot copper grids, stained with uranyl acetate and lead citrate, and examined in a JEM 1010 transmission electron microscope (JEOL, USA, Inc.) at an accelerating voltage of 80 kV. Digital images were obtained using an AMT Imaging System (Advanced Microscopy Techniques).

### Confocal microscopy and image processing.

Confocal imaging was performed using a Leica TCS SP8 laser scanning confocal microscope equipped with an acousto-optical beam splitter. Multiprobe fluorescence imaging with *Z*-stacking was performed through sequential bidirectional scanning using between-lines acquisition mode, 600 Hz scan speed, 3,000 × 3,000 format, and optimized z-step sizes. For crystals imaging, a separate channel was configured for reflection microscopy and scanned sequentially with fluorescence acquisition channels. The reflection imaging channel used a photomultiplier tube detector, with the acousto-optical beam splitter configured to reflection mode. An 458 nm argon laser was used for incident light, and detection wavelength range was optimized to 455–463 nm. Image *Z*-stacks were projected onto 2D composite images using the maximum intensity projection method. Image processing and quantification were conducted using ImageJ software. Acquired images were converted to 8 bit grayscale, and background noise was mitigated using a median filter with a pixel radius of 2.0. For particle analysis and counting, images were segmented via thresholding and converted to binary. Cells counts were obtained via nuclei quantification within segmented cell masks.

### Brain nuclei isolation.

Anesthetized mice were transcardially perfused with 40 mL of PBS. After perfusion, an 8–12 mm^3^ sample of brain tissue containing corpus callosum adjacent was excised and minced into pieces approximately 1 mm^3^ on wet ice per mouse. Minced tissue was transferred to prechilled glass Dounce homogenizers with homogenization buffer composed of 250 mM sucrose, 25 mM KCl, 5 mM MgCl_2_, 1.0 μM DTT, and 0.1% TritonX100 in 10 mM Tris buffer, pH 8.0, supplemented with RNase (0.4 U/μL RNasin Promega and 0.2 U/μL SUPERaseIn Invitrogen) and protease (1× complete cocktail Roche) inhibitors and nuclei acid dyes (1.0 μg/mL PI and 1.0 μg/mL DAPI). Tissue was homogenized on wet ice using a loose-fitting pestle for 5 strokes and then a tight-fitting pestle for 15 strokes. Homogenate was filtered through 50 μm strainers into low-DNA-binding microtubes. Nuclei were pelleted at 400*g* at 4°C for 5 minutes and resuspended in isolation buffer consisting of 1 mg/mL BSA in nuclease free DPBS supplemented with RNase (0.4 U/μL RNasin, Promega; and 0.2 U/μL SUPERaseIn, Invitrogen) and protease (1× complete cocktail, Roche) inhibitors and nuclei acid dyes (1.0 μg/mL propidium iodide [PI] and 1.0 μg/mL DAPI). Nuclei were filtered through a 40 μm strainer and isolated from debris via fluorescence-activated nuclei sorting using singlet and DAPI^+^, PI^+^ dual gating, achieving an estimated nuclei concentration of 900–1,000 nuclei/μL.

### Single-nuclei RNA-Seq.

snRNA-Seq libraries were constructed in tandem using the 10x Genomics Chromium Single Cell 3′ system (version 3.1; dual index) per manufacturer’s instructions. Dual-indexed libraries were sequenced using NovaSeq 6000 (Illumina) with 150 bp paired-end sequencing at a target depth of 35,000 reads per nucleus and target sample size of 9,000 nuclei per library. Demultiplexed raw reads were filtered and aligned to the reference genome (mm10) using Cell Ranger (version 7.0.0) count with intron reads included (10x Genomics) (61). Per the library, ambient RNA contamination was scrubbed using decontX (celda, version 1.22.1) (62). Low-quality transcriptomes were filtered out using gene count and mitochondrial read cutoffs of 200 and 5.0%, respectively. Genes detected in fewer than 10 cells were excluded from analysis.

Gene counts were then normalized and scaled using the Seurat LogNormalize method followed by scaling (Seurat, version 5.2.1) (63). Dimensionality reduction and cell clustering were achieved using PCA and the Seurat shared nearest neighbor (SNN) clustering algorithm, respectively (Seurat, version 5.2.1) (63). Doublets were detected for each dataset individually via scDblFinder (scDblFinder, version 1.20.2) using an estimated doublet rate of 1.0% per 1,000 nuclei detected (64). After excluding doublets, processed datasets were aggregated into a single library.

Normalization, dimensionality reduction, and clustering were repeated for the aggregated library using SCTransform (65), PCA, and the Seurat SNN clustering algorithm, respectively (Seurat, version 5.2.1) (63). DEGs per cluster were identified via the Wilcoxon rank sum test using FindAllMarkers (Seurat, version 5.2.1), with log_2_ fold change and percentage-expressed thresholds of 0.25 and 25%, respectively. For pseudobulk analysis, a matrix of gene counts across samples was generated by summing the corrected unique molecular identifier counts per gene across all cluster-annotated cells per sample. DEG analysis was performed on the generated gene count matrices using DESeq (DESeq2, version 1.46.0) (60).

### Pathway analysis.

Kyoto Encyclopedia of Genes and Genomes (KEGG) pathway analysis was performed via overrepresentation analysis with 1-sided Fisher’s exact test using enrichKEGG (clusterProfiler, version 4.14.6) (66). Overrepresentation analysis was performed using upregulated (adjusted *P* < 0.01, log_2_ fold change > 0.25) and downregulated (adjusted *P* < 0.01, log_2_ fold change < –0.25) DEGs to determine enriched and reduced pathways, respectively. Background genes used in pathway analysis among DMAM2, DMAM1, and DMAM0 clusters required a minimum average expression of 0.5 within the DMAM0 cluster. Background genes used in bulk RNA-Seq pathway analysis required a minimum average expression of 10 across libraries. Minimum and maximum cutoffs for pathway sizes were 10 and 300, respectively. For bulk RNA-Seq, upstream regulator activity was inferred from DESeq analysis–derived fold change using Qiagen IPA suite (67). Thresholds for statistically significant changes in upstream regulator activity included adjusted *P* value and *z* score cutoffs of 0.05 and |2.0|, respectively.

### Enrichment analysis of customized gene sets in bone marrow–derived macrophages.

Customized gene sets were generated using annotated microglial cluster marker genes from published snRNA-Seq datasets (68, 69). Gene sets were generated by selecting up to 100 top marker genes (ranked by *P* value) corresponding to annotated clusters. Enrichment of gene sets in PEX5cKO relative to controls was assessed through gene set enrichment analysis using clusterProfiler (version 4.14.6) and gene lists ranked by fold change. An adjusted *P* value of 0.05 was the cutoff for statistically enriched (normalized enrichment score >0) or decreased (normalized enrichment score <0) gene sets in PEX5cKO relative to controls.

### Trajectory analysis.

Developmental trajectories were inferred using slingshot (version 2.14.0) (17). Low-dimensional space coordinates from uniform manifold approximation and projection and cluster annotations were inputted into slingshot to map connections between adjacent clusters using a minimum spanning tree and to infer lineages across mapped connections. The initial clusters within microglial and oligodendrocyte lineages were specified based on prior knowledge using established gene markers for homeostatic microglia and committed oligodendrocyte precursors, respectively. Smooth lineage curves were generated using a resolution of up to 400 cells.

### Statistics.

Statistical analysis was conducted in R. An unpaired, 2-tailed *t* test was used for comparisons between 2 groups. The FDR method was used for multiple testing correction. For comparison between multiple groups, 1-way ANOVA was conducted followed by Tukey honestly significant difference (HSD) for post hoc testing. *P* < 0.05 was considered significant. The number of animals or wells used per experiment are indicated in the corresponding figures. For immunoblotting and real-time qPCR, representative data from multiple independent replicates are shown. No statistical methods were used to predetermine sample size. No blinding or randomization was implemented during experiments.

### Study approval.

Mouse work was conducted in accordance with protocols approved by the IACUC of the University of Texas MD Anderson Cancer Center.

### Data availability.

Underlying data for the figures presented in the main text and supplemental material are available in the Supporting Data Values file. Raw and processed data for bone marrow–derived macrophage bulk RNA-Seq and mouse brain single-cell RNA-Seq are available in the Gene Expression Omnibus repository within the GSE255159 and GSE272138 series, respectively. The analytic code used for RNA-Seq analysis is publicly available on GitHub at https://github.com/loui-kwam/PEX5 (GitHub ID: loui-kwam).

## Author contributions

JABV contributed to the study conception and design, optimization and conduction of experiments, data analysis and interpretation, and manuscript drafting and editing. YLPS assisted with and performed immunofluorescence sample preparation and immunoblotting. KAS assisted with and performed immunofluorescence sample preparation, image acquisition, and analysis. YG and XZ assisted with animal husbandry and provided support during experiments. JH contributed to the study conception and design, data interpretation, and manuscript editing.

## Funding support

This work is the result of NIH funding, in whole or in part, and is subject to the NIH Public Access Policy. Through acceptance of this federal funding, the NIH has been given a right to make the work publicly available in PubMed Central.

- NIH (grants F31NS124110 and R01NS127933).
- MD Anderson Cancer Neuroscience Program internal fund.
- Cancer Center Support grant from the NIH (P30CA016672).

## Supplementary Material

Supplemental data

Unedited blot and gel images

Supplemental table 1

Supplemental table 2

Supplemental table 3

Supplemental table 4

Supplemental table 5

Supplemental table 6

Supplemental table 7

Supplemental table 8

Supporting data values

## Version 1 11/06/2025

In-Press Preview

## Version 2 01/02/2026

Electronic publication
