# Supplementary material for: Peroxisomal integrity in demyelination-associated microglia enables cellular debris clearance and myelin renewal in mice
Source: J Clin Invest. 2025 Nov 6;136(1):e179985. doi: 10.1172/JCI179985 (PMC12721888; doi:10.1172/JCI179985)
Supplement: Supplemental data [file jci-136-179985-s077.pdf]

Supplemental Tables

Supplemental Table 1: Gene markers for major cell types detected in brain specimens collected from control and PEX5cKO mice at baseline and CPZ conditions.

Supplemental Table 2: Gene markers for immune sub-clusters detected in brain specimens collected from control and PEX5cKO mice at baseline and CPZ conditions.

Supplemental Table 3: Differentially expressed genes with adjusted p value <0.15 detected from pseudobulk analysis between PEX5cKO and control genotypes across immune sub-clusters within the CPZ condition.

Supplemental Table 4: Result tables from differentially expressed gene analysis performed between DMAM2 and DMAM0 clusters and between DMAM2 and DMAM1 clusters.

Supplemental Table 5: Differentially expressed genes (adjusted p value <0.01, absolute fold change >0.25, base mean expression >10) detected between PEX5cKO and control bone marrow-derived macrophages across resting, 24h, and 72h conditions.

Supplemental Table 6: Marker genes of annotated microglia clusters from published single cell RNA sequencing datasets used to generate custom gene signatures for gene set enrichment analysis in bone marrow-derived macrophages

Supplemental Table 7: Gene markers for OPC and oligodendroglial lineage sub-clusters detected in brain specimens collected from control and PEX5cKO mice at baseline and CPZ conditions.

Supplemental Table 8: Differentially expressed genes with adjusted p value <0.15 detected from pseudobulk analysis between PEX5cKO and control genotypes across oligodendroglia lineage sub-clusters within the CPZ condition.

Supplemental Figures and Figure Legends

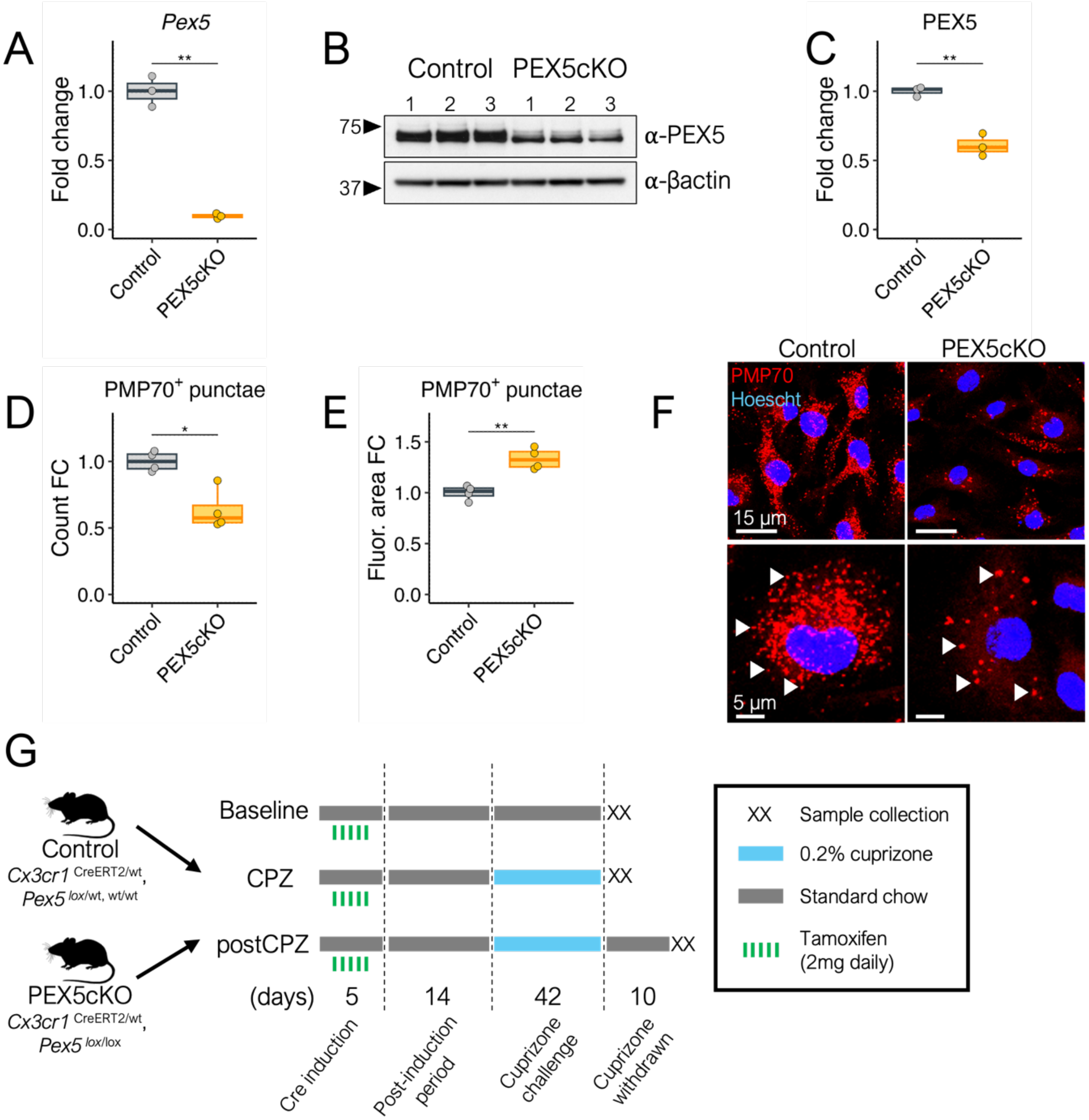

Supplement Figure 1: Validation of PEX5 depletion and experimental design for conditional knockout in vivo. (**A**) *Pex5* transcript fold change relative to control mean expression in bone marrow-derived macrophages. (**B**) Representative PEX5 and βactin antibody immunoblots using whole cell lysates from bone marrow-derived macrophages. Lanes correspond to biological replicates. (**C**) PEX5 fold change relative to control mean in bone marrow-derived macrophages.

Datapoints correspond to biological replicates. (**D**) Fold change of PMP70$^+$ punctae count per cell relative to control mean in bone marrow-derived macrophages. Datapoints correspond to biological replicates. (**E**) Fold change of average PMP70$^+$ puncta area fluorescence relative to control mean in bone marrow-derived macrophages. Datapoints correspond to biological replicates. (**F)** Representative confocal micrographs for PMP70 (red) immunofluorescence of bone marrow-derived macrophages. Nuclei are stained with Hoescht. Arrow heads highlight representative PMP70$^+$ punctae. Top scale bars = 15 µm, bottom scale bars = 5 µm. (**G**) Schematic demonstrating experimental design to derive control and PEX5cKO samples for baseline, CPZ, and postCPZ conditions. For (**A**, **C**, **D**, and **E**) statistical analysis involved unpaired, two tailed t test, *$p<0.05$, **$p<0.01$. CPZ, cuprizone-fed, postCPZ, post cuprizone removal.

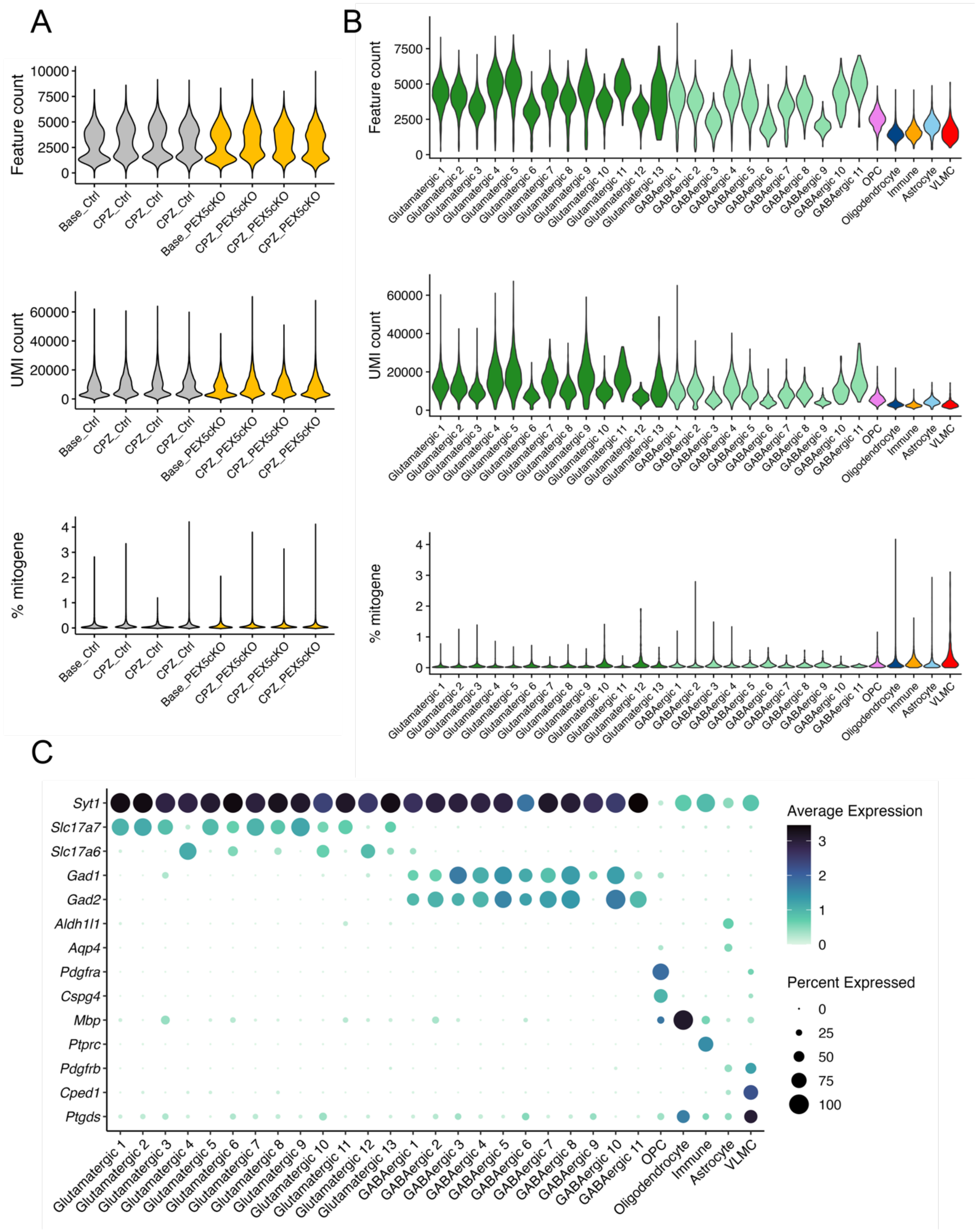

A
Feature count
UMI count
% mitogene
Base_Ctrl
CPZ_Ctrl
CPZ_Ctrl
CPZ_Ctrl
Base_PEX5cKO
CPZ_PEX5cKO
CPZ_PEX5cKO
CPZ_PEX5cKO
B
Feature count
UMI count
% mitogene
Glutamatergic 1
Glutamatergic 2
Glutamatergic 3
Glutamatergic 4
Glutamatergic 5
Glutamatergic 6
Glutamatergic 7
Glutamatergic 8
Glutamatergic 9
Glutamatergic 10
Glutamatergic 11
Glutamatergic 12
Glutamatergic 13
GABAergic 1
GABAergic 2
GABAergic 3
GABAergic 4
GABAergic 5
GABAergic 6
GABAergic 7
GABAergic 8
GABAergic 9
GABAergic 10
GABAergic 11
OPC
Oligodendrocyte
Immune
Astrocyte
VLMC
C
Syt1
Slc17a7
Slc17a6
Gad1
Gad2
Aldh1l1
Aqp4
Pdgfra
Cspg4
Mbp
Ptprc
Pdgfrb
Cped1
Ptgds
Average Expression
Percent Expressed
0
25
50
75
100

Supplement Figure 2: Single nuclei RNA sequencing library quality metrics and cell clusters identified. (**A**) Distribution of feature count, UMI count, and mitogene percentage per cell within each single nuclei RNA sequencing library generated. (**B**) Distribution of genes detected, UMI count, and mitogene percentage per cell across detected cell clusters from aggregated single nuclei RNA sequencing library. (**C**) Average gene expression of cell cluster marker genes in aggregated single nuclei RNA sequencing library. Dot color and size correspond to average expression and percent expressed, respectively. UMI, unique molecular identifier, single nuclei RNA sequencing, single nuclei RNA sequencing.

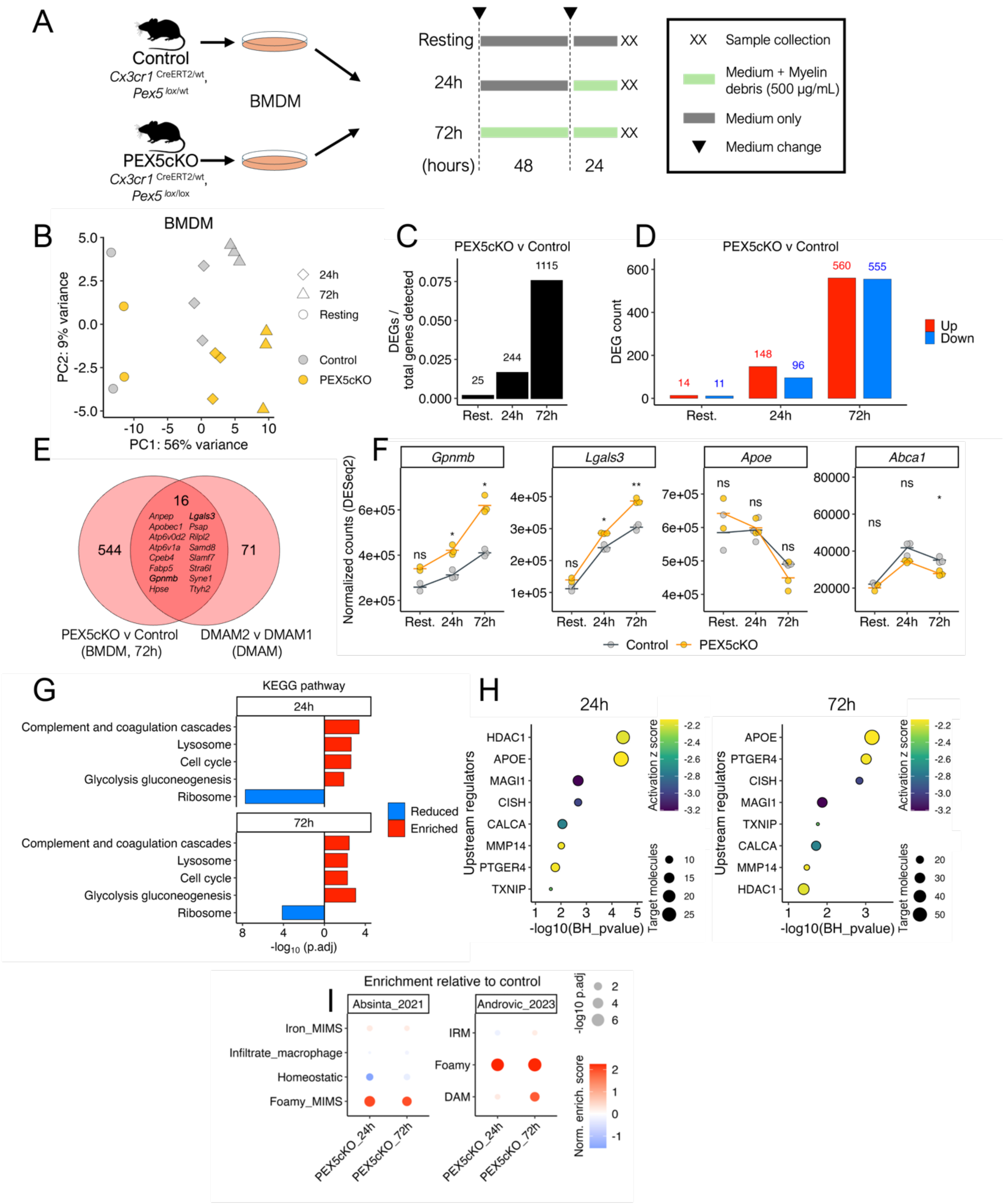

Supplement Figure 3: Myelin debris exposure in bone marrow-derived macrophages partially reproduces DMAM2 signature. (**A**) Schematic demonstrating experimental design to

generate control and PEX5cKO bone marrow-derived macrophages under resting, 24h, and 72h conditions. (**B**) Principal component analysis plot for control and PEX5cKO bone marrow-derived macrophages at resting, 24h, and 72h conditions. (**C**) Ratio of DEGs to total genes detected per condition. Numbers over bars indicate total DEG count >0 per condition. (**D**) Upregulated (Up) or downregulated (Down) DEG counts per condition. Numbers over bars indicate DEG count >0 per condition. (**E**) Venn diagram triangulating DEGs upregulated in DMAM2 subcluster (87) and in PEX5cKO bone marrow-derived macrophages (560). (**F**) Normalized counts for indicated RNA transcripts across conditions colored according to genotype. Bars and datapoints correspond to mean and individual biological replicates. Unpaired, two tailed t test between genotypes across conditions, ns $p>0.05$, *$p<0.05$, **$p<0.01$. (**G**) Enriched (red, $-\log_{10}$p.adj >1.3, overrepresented in upregulated DEGs) and repressed (blue, $-\log_{10}$p.adj >1.3, overrepresented in downregulated DEGs) KEGG pathways shared between 24h and 72h conditions. (**H**) Upstream regulators with repressed activity ($-\log_{10}$BH_p >1.3, activation z score ≤ -2.0) in PEX5cKO BMDM relative to control at 24h and 72h conditions. (**I**) Enrichment analysis for molecular signatures in PEX5cKO bone marrow-derived macrophages post myelin debris exposure relative to control. Dot color and size correspond to NES and $-\log_{10}$p.adj, respectively. Signatures non-enriched ($-\log_{10}$p.adj <1.3) in PEX5cKO macrophages are opaque. Molecular signatures are derived from previously reported single cell RNA sequencing datasets of mouse (Androvic et al., 2023) and human (Absinta et al., 2021) brain.

BMDM, bone marrow-derived macrophages, KEGG, Kyoto Encyclopedia of Genes and Genomes, Resting, myelin debris free, 24h, myelin debris exposure for 24 hours, 72h, myelin debris exposure for 72 hours, NES, normalized enrichment score, BH, Benjamini-Hochberg corrected, ns, not significant.

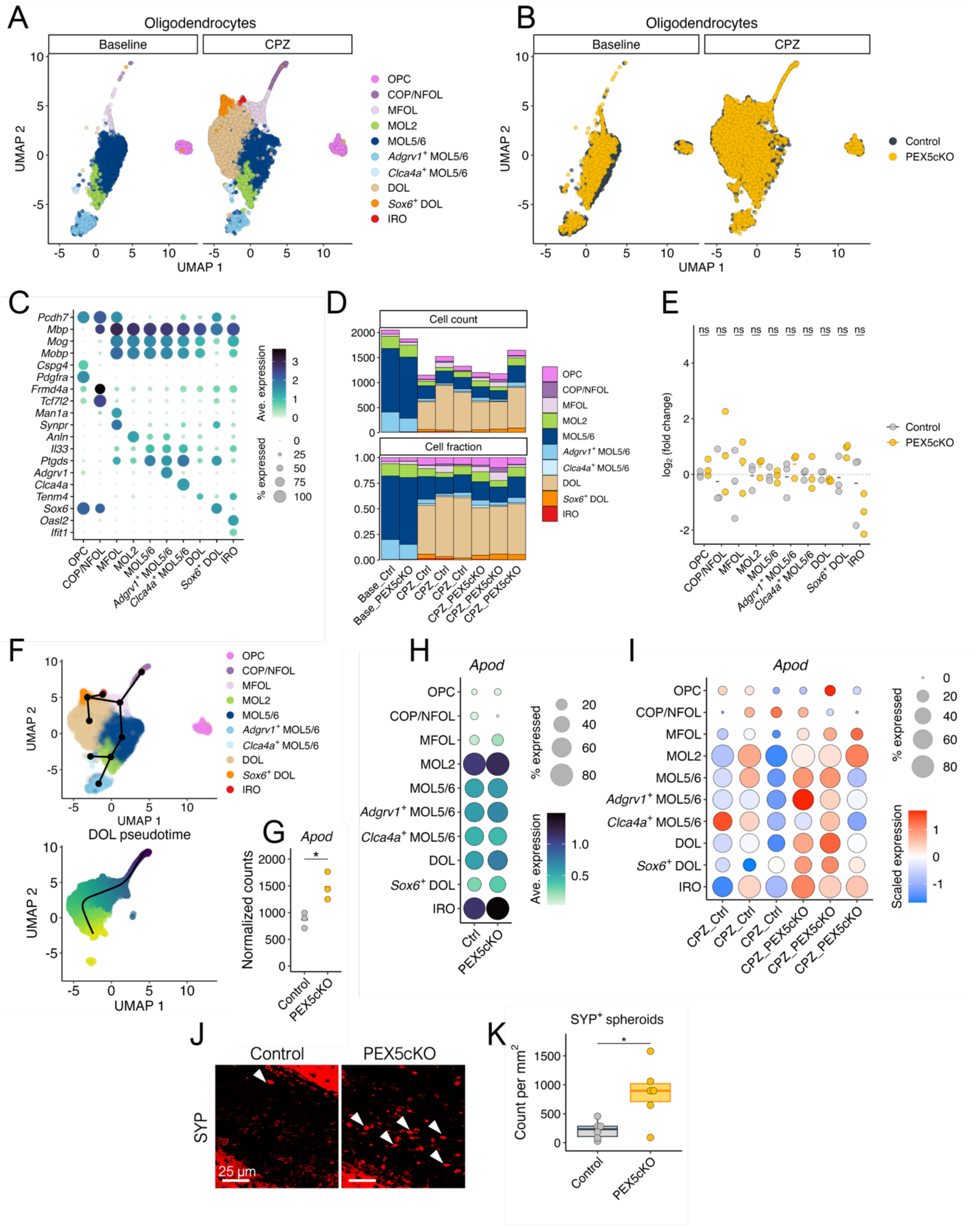

Supplement Figure 4: PEX5cKO DMAMs associated with *Apod* upregulation in demyelination associated oligodendrocytes and elevated axonal damage. (**A**) UMAP of

oligodendrocyte lineage colored according to cluster identity and split by conditions. (**B**) UMAP of oligodendrocyte lineage colored according to genotype and split by condition. (**C**) Average gene expression of marker genes per oligodendrocyte lineage clusters. Dot color and size correspond to average expression and percent expressed, respectively. (**D**) Cell count and cell fraction per oligodendrocyte lineage cluster identified within each library generated across baseline (n=1 per genotype) and CPZ (n=3 per genotype) conditions. (**E**) Log_2 fold change relative to control of the cell fractions per oligodendrocyte lineage cluster. Individual datapoints corresponds to biological replicates collected from the CPZ condition. Bars correspond to the mean log_2 fold change. Two tailed t test, ns $p>0.05$. (**F**) (Top) Inferred lineages for oligodendrocyte clusters derived from slingshot analysis superimposed onto oligodendrocyte lineage UMAP, with the COP/NFOL subcluster designated as the origin cluster. (Bottom) Pseudotime trajectory for the DOL lineage. (**G**) DESeq2-mediated normalized pseudobulk counts for *Apod* gene within the DOL cluster detected within the CPZ condition. Two tailed t test, $^*p<0.05$. Individual datapoints correspond to biological replicates. Bars correspond to normalized pseudobulk count mean. (**H**) Average gene expression of *Apod* per oligodendrocyte lineage cluster detected across genotypes. Dot color and size correspond to average gene expression and percent expressed, respectively. (**I**) Average gene expression of *Apod* scaled across biological replicates per oligodendrocyte lineage cluster detected across genotypes within the CPZ condition. Dot color and size correspond to scaled gene expression and percent expressed, respectively. (**J**) Representative confocal micrographs for SYP^+ immunofluorescence within the corpus callosum of control and PEX5cKO mice collected in the postCPZ condition. Arrowheads indicated representative SYP^+ spheroids. Scale bar = 25 μm. (**K**) SYP^+ spheroid count per mm^2 in control and PEX5cKO mice collected in the postCPZ condition. Individual datapoints correspond to biological replicates. Two tailed t test, $^*p$ value <0.05.

SYP, synaptophysin, UMAP, uniform manifold approximation and projection, OPC, oligodendrocyte progenitor cell, COP/NFOL, committed oligodendrocyte progenitor/newly formed oligodendrocyte, MFOL, myelin forming oligodendrocyte, MOL5/6, mature oligodendrocyte subtype 5/6, MOL2, mature oligodendrocyte subtype 2, DOL, demyelination associated oligodendrocyte, IRO, interferon responsive oligodendrocyte, CPZ, cuprizone-fed, ns, not significant.
