# Supplementary figures and images for: Peroxisomal integrity in demyelination-associated microglia enables cellular debris clearance and myelin renewal in mice
Source: J Clin Invest. 2025 Nov 6;136(1):e179985. doi: 10.1172/JCI179985 (PMC12721888; doi:10.1172/JCI179985)

Full unedited blots for Supplemental Figure 1B.

PEX5

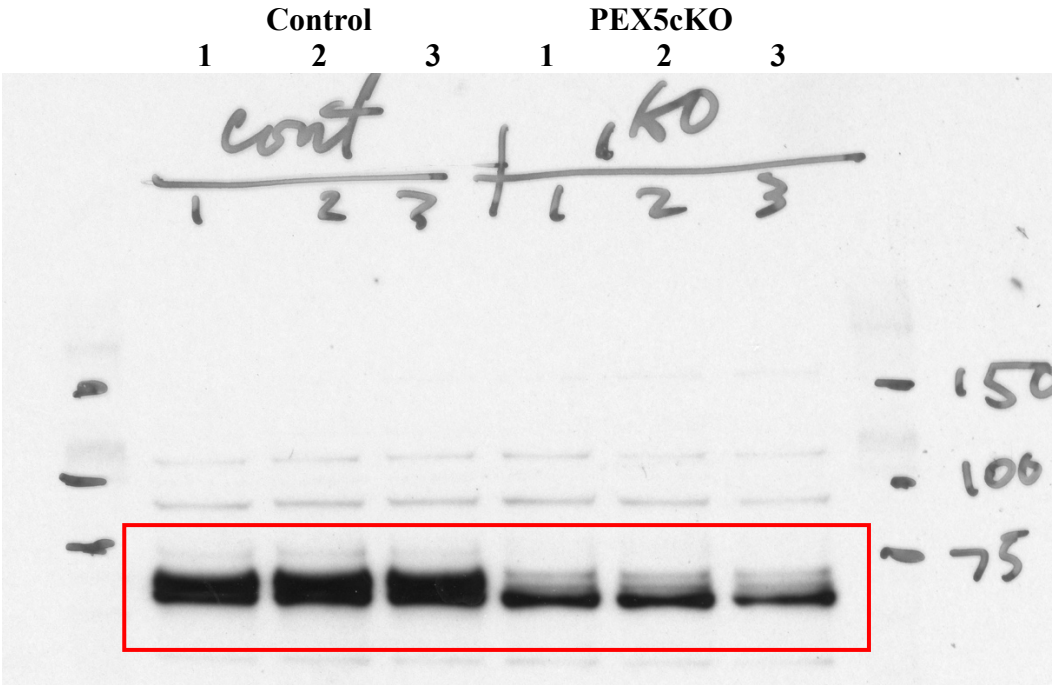

Beta-actin

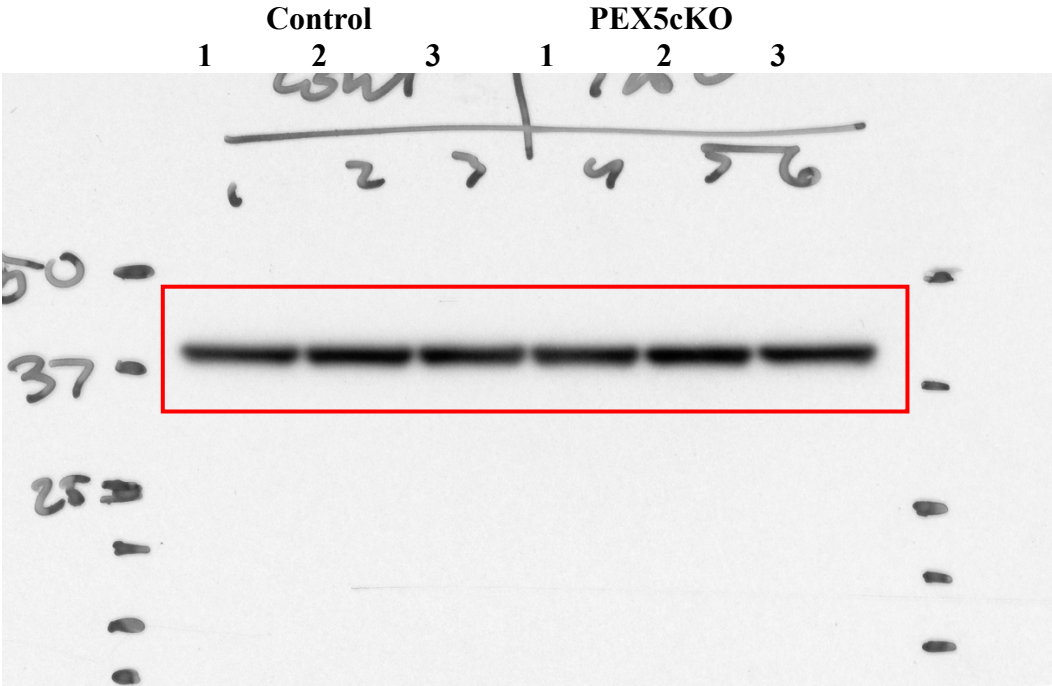

Supplement: Unedited blot and gel images [file jci-136-179985-s078.pdf]
